# Dietary Patterns and Nutritional Interventions for Cognitive Impairment in Parkinson’s Disease: A Critical Narrative Review

**DOI:** 10.3390/nu18203436

**Published:** 2026-10-20

**Authors:** Amaal Aldakheel, Najeeb Qadi

**Affiliations:** 1 Neuroscience Centre of Excellence, King Faisal Specialist Hospital and Research Centre, Riyadh 11211, Saudi Arabia; 2 College of Medicine, Alfaisal University, Riyadh 11533, Saudi Arabia

**Keywords:** Parkinson’s disease, mild cognitive impairment, dementia, Mediterranean diet, ketogenic diet, dietary supplements, vitamin D, probiotics, saffron, cognitive outcomes, narrative review

## Abstract

Background/Objectives: Cognitive impairment affects most people with Parkinson’s disease (PD); mild cognitive impairment is present in 25–40% of people with PD without dementia, and nutritional advice often exceeds the evidence. We reviewed the human evidence that dietary patterns or nutritional supplements improve cognition in established PD, separating objective cognitive outcomes from patient-rated and biomarker outcomes. Methods: Critical narrative review reported according to SANRA. Sources were PubMed, Embase, Web of Science and Cochrane CENTRAL (inception to 29 September 2026) and reference lists. Controlled intervention studies in established idiopathic PD with an objective cognitive outcome or a specifically rated cognitive symptom formed the evidence set, appraised on risk-of-bias domains and classified by an explicit rule. Results: Twenty-three randomized controlled studies met the criteria (five dietary patterns, 18 supplements). Favorable between-group differences were reported in six studies (Mediterranean diet, a ketogenic pilot, creatine–coenzyme Q10, a probiotic, saffron and ginkgo leaf extract), all with fewer than 100 participants and heterogeneous cognitive endpoint status. The three largest trials did not demonstrate cognitive benefit. Other reports showed nonsignificant comparisons or did not provide a between-group cognitive estimate; one used only a rated symptom. No study showed durable functional benefit or reduced progression to dementia. Conclusions: No nutritional intervention has independently replicated, durable cognitive efficacy in PD. Nutritional adequacy, correction of documented deficiency, dysphagia care and individualized levodopa–meal timing remain the clinical priorities; a Mediterranean-style pattern is a reasonable default, not a cognitive treatment.

## 1. Introduction

Parkinson’s disease (PD) affected an estimated 11.8 million people in 2021, and the number living with the disease is projected to reach 25 million by 2050 [1,2]. Cognitive impairment is one of its most disabling non-motor features. In a pooled analysis of 1346 people with PD without dementia, 25.8% met criteria for PD mild cognitive impairment (PD-MCI) [3], and a meta-analysis of 41 studies that applied the Movement Disorder Society (MDS) criteria estimated a pooled prevalence of 40% [4]. In incident cohorts, 46% developed dementia within 10 years, and 83% of those followed for 20 years had dementia [5,6]. PD-MCI is heterogeneous and dynamic: about 39% of people with PD-MCI progress to PD dementia (PDD) within five years, whereas about one in four revert to normal-range performance [7,8]. Formal criteria define PD-MCI as objective impairment with preserved functional independence and PDD as cognitive decline that interferes with everyday function [9,10,11]. These distinctions matter for nutritional research: a change on a brief screening instrument, a domain-specific neuropsychological change and conversion to PDD are different outcomes.

PD is an α-synuclein proteinopathy. Cognitive impairment in PD reflects the spread of Lewy pathology to limbic and cortical regions, often with coexisting Alzheimer-type and vascular pathology and with cholinergic and dopaminergic deficits, and it co-occurs with motor, autonomic and neuropsychiatric impairment that itself affects daily function and test performance [11]. Because Lewy pathology may begin in the enteric nervous system and olfactory bulb, and because PD risk varies with environmental exposures, dietary habits have been studied both as risk factors for onset and as modifiable influences after diagnosis [12,13].

Nutrition influences vascular and metabolic health, energy balance, gastrointestinal function, medication absorption and micronutrient status [14,15]. Observational studies associate Mediterranean and MIND (Mediterranean–DASH Intervention for Neurodegenerative Delay) patterns with lower PD incidence, fewer prodromal features and later onset [13,16,17,18,19]. Previous reviews have summarized dietary and supplement interventions across PD outcomes, emphasizing limited, heterogeneous evidence [20,21,22,23,24,25,26]. Cognition-specific synthesis includes Mediterranean-diet evidence from a single trial [27], while biomarker meta-analyses associate homocysteine, vitamin B12, folate and vitamin D with cognitive performance [28,29]. These findings warrant appraisal focused on cognition in established PD.

Such appraisal must distinguish prevention from treatment, objective cognition from patient ratings and biomarkers, and association from efficacy [12,26]. Three therapeutic claims require separate evaluation: nutritional rescue, restoring function lost through inadequate intake or deficiency; cognitive enhancement, improving performance in nutritionally replete patients; and disease modification, slowing decline or reducing progression to PDD. These claims require different populations, endpoints and follow-up durations.

The question is clinically pressing. Patients and families ask about diet and supplements at most visits, and restrictive regimens carry real risk in a population prone to weight loss, dysphagia, malnutrition and sarcopenia [30,31,32,33]. Supplements add cost and interaction burden. Clinicians therefore need an evidence-calibrated answer that states what is known, what remains unproven, and what remains appropriate care regardless.

This review asks what human evidence supports a cognitive benefit from dietary patterns or nutritional interventions in established PD. It prioritizes controlled studies with cognitive outcomes, distinguishes objective testing from patient ratings and biomarkers, and separates nutritional rescue, short-term performance and progression. Dietary patterns and supplements are appraised by study design, endpoint hierarchy and clinical relevance. PD-risk cohorts and studies limited to motor or gastrointestinal outcomes provide context. The aim is an evidence-calibrated interpretation for the PD-MCI clinic.

## 2. Materials and Methods

### 2.1. Design and Reporting

This is a critical narrative review, not a systematic review or meta-analysis, and no protocol was registered. Reporting follows the domains of the Scale for the Assessment of Narrative Review Articles (SANRA): justification of importance, a concrete aim, description of information sources, appropriate referencing, scientific reasoning and presentation of endpoint data [34]. SANRA rates the review itself, not the trials it contains; study-level appraisal is described in Section 2.5. The search, selection and extraction are reported with the transparency expected of a systematic review, but the review does not claim exhaustive coverage.

### 2.2. Information Sources and Search

Two retrieval stages were used. For the original submission, English-language human reports were identified by targeted retrieval that combined PD, cognitive outcomes and named dietary or nutritional interventions in PubMed-indexed records, publisher pages and DOI-based bibliographic records, with reference-list checking of the included studies and of two previous reviews [25,26]; the publication cutoff was 31 August 2026. Named interventions were selected because they had been tested in at least one controlled study in PD with a cognitive outcome, or because clinicians are commonly asked about them (Mediterranean, MIND and ketogenic diets, protein redistribution, vitamins D and B12, folate, creatine, coenzyme Q10 (CoQ10), omega-3 fatty acids, probiotics, saffron and combined metabolic activators).

**Figure 1:**
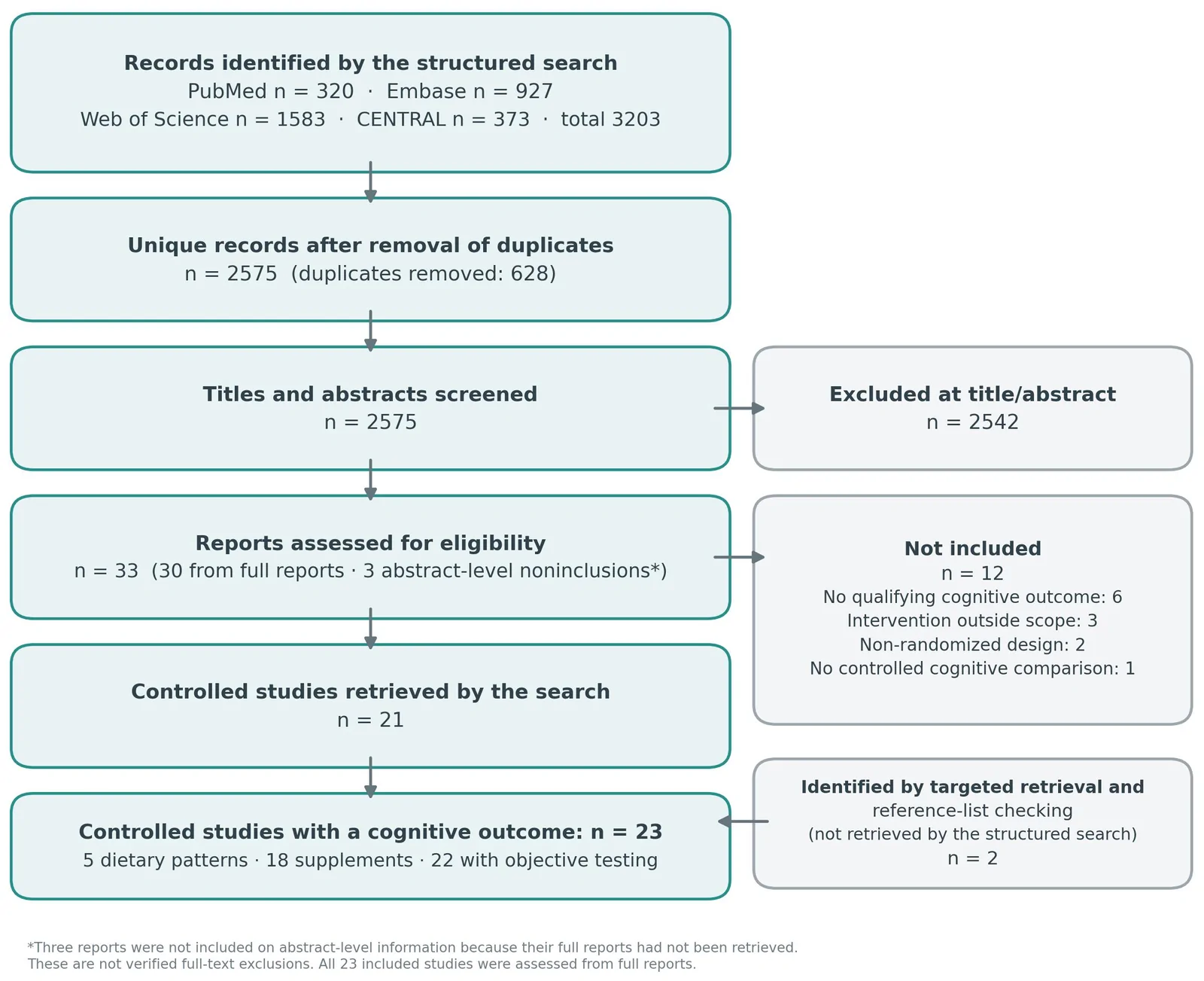
Flow of records for the structured search (29 September 2026), incorporating proof-stage full-report verification on 19 October 2026. Of 33 reports selected for assessment, 30 were assessed from full reports (21 included and nine not included). Mehrabani 2023 remained outside the controlled cognitive evidence set after full-report assessment because no eligible cognitive treatment outcome was reported [35]. Three additional reports (Zali 2024, Cesarone 2020 and Belcaro 2022) were not included on the basis of abstract-level information because their full reports had not been retrieved [46,47,48]. These three are not represented as verified full-text exclusions. The 21 included reports from the search and two full reports identified by targeted retrieval (NET-PD LS-1 [49] and the KIM trial [50]) give 23 included studies, all assessed from full reports. Chong’s randomized three-month phase is included; its uncontrolled follow-up is not [51].

For this revision, a structured search was run on 29 September 2026 in PubMed, Embase (embase.com), Web of Science Core Collection and the Cochrane Central Register of Controlled Trials (CENTRAL) through the King Faisal Specialist Hospital and Research Centre library, from each database’s inception, limited to human studies in English. Four concept blocks were combined: Parkinson’s disease; cognition or cognitive outcomes; dietary pattern, food-based or nutritional-supplement intervention; and controlled intervention design. In abbreviated form, the PubMed string was: (“Parkinson Disease”[Mesh] OR parkinson*[tiab]) AND (“Cognition”[Mesh] OR “Neurocognitive Disorders”[Mesh] OR cognit*[tiab] OR dementia[tiab] OR “mild cognitive impairment”[tiab] OR MoCA[tiab] OR MMSE[tiab]) AND (“Diet”[Mesh] OR “Dietary Supplements”[Mesh] OR diet*[tiab] OR nutrition*[tiab] OR supplement*[tiab] OR ketogenic[tiab] OR Mediterranean[tiab] OR vitamin*[tiab] OR creatine[tiab] OR “coenzyme Q10”[tiab] OR probiotic*[tiab] OR saffron[tiab]) AND (“Randomized Controlled Trial”[pt] OR randomized[tiab] OR placebo[tiab] OR “controlled trial”[tiab] OR “control group”[tiab]) NOT (animals[mh] NOT humans[mh]) AND english[la]. The complete strings for all four databases, with the full term lists, are given in Supplementary S1. The search retrieved 320 records from PubMed, 927 from Embase, 1583 from Web of Science and 373 from CENTRAL (3203 in total; 2575 unique records after removal of duplicates by DOI, PubMed identifier and normalized title). Unique records were pre-sorted by keyword rules applied to the title and abstract (Supplementary S1) and then screened by title and abstract by one author (N.Q.); 2542 were excluded at that stage, and 33 reports were selected for further assessment. All 33 have recorded dispositions: 21 included and 12 not included in the current evidence set. Thirty of the 33 were assessed from full reports (21 included and nine not included). This incorporates proof-stage verification of the full report by Mehrabani et al. [35] on 19 October 2026; the report remained outside the controlled cognitive evidence set because no eligible cognitive treatment outcome was reported. Three additional reports were not included on the basis of abstract-level information because their full reports had not been retrieved; these are not represented as verified full-text exclusions. Figure 1 displays these routes. Of the 13 controlled studies in the original submission, 11 were retrieved by the structured search; the NET-PD LS-1 report and the KIM trial were not, because neither abstract contains a cognition term, and had been identified by the original targeted retrieval and reference-list checking. The structured search also identified nine randomized controlled studies with an objective cognitive outcome that the original retrieval had missed: the DATATOP tocopherol comparison [36,37], a 12-month vitamin D trial [38], a niacin trial [39], three probiotic trials [40,41,42], a fermented papaya crossover trial [43], and trials of ginkgo leaf tablets [44] and boswellic acids [45]. Final eligibility adjudication also retained the randomized three-month phase of an additional niacin trial, described in Section 5.1, while excluding its later uncontrolled follow-up from the controlled evidence set. Together, these ten additions give 23 controlled studies. All 23 included studies were assessed from full reports. DATATOP is represented by the 1993 primary report in the *New England Journal of Medicine* and the 1994 cognitive brief communication in *Neurology*, linked as reports of one trial [36,37]. Reference lists of included studies and of the reviews cited above were hand-searched. Trial registries and grey literature were not searched. Figure 1 shows the flow of records.

### 2.3. Eligibility Criteria

Table 1 gives the eligibility boundary. The controlled evidence set required (a) adults with established idiopathic PD at any stage, with or without PD-MCI or PDD; (b) a dietary pattern, food-based or nutritional-supplement intervention; (c) a concurrent comparison group with random or quasi-random allocation; (d) at least one objective cognitive outcome, defined as a standardized neuropsychological test or screening instrument, or a specifically rated cognitive symptom reported separately from global or motor scores; quality-of-life subscales that name cognition (for example the PDQ-39 cognitive-impairment dimension) do not qualify; and (e) publication as a full peer-reviewed report in English rather than a conference abstract. All included studies were assessed from their full reports. These boundaries were applied during revision, not in a preregistered protocol. Reports judged outside this scope were retained only as context. Non-randomized controlled studies were treated as context because allocation by physician or patient choice in open supplement registries does not support causal inference; two such studies of Pycnogenol with physician-rated cognitive symptom scores were identified by the structured search and are cited in Section 5.6 [46,47]. The Romanian open-label vitamin pilot [52] illustrates the uncontrolled category: it informs clinical interpretation but has no comparison group.

**Table 1:** Eligibility boundary for the controlled evidence set and the contextual evidence.

Controlled Evidence Set (Counted; Table 2 and Table 3)	Contextual Evidence (Not Counted; Discussed Where Relevant)
Adults with established idiopathic PD at any stage, with or without PD-MCI or PDD	PD-risk, prodromal or prevention cohorts without established PD
A dietary pattern, food-based or nutritional-supplement intervention (diet; micronutrient; mitochondrial or metabolic nutrient; probiotic or synbiotic; botanical food supplement; omega-3; MCT or ketone product)	Pharmacological agents, exercise or cognitive training, even when combined with diet
A concurrent comparison group (placebo, usual diet or active comparator) with random or quasi-random allocation	Non-randomized controlled studies (for example the two Pycnogenol registry studies [46,47]) and uncontrolled before–after studies, for example the Romanian open-label vitamin pilot [52]
At least one objective cognitive outcome (standardized neuropsychological test or screening instrument) or a specifically rated cognitive symptom reported separately from global or motor scores	Biomarker-only or mechanistic outcomes; reports with motor, gastrointestinal or quality-of-life outcomes and no cognitive outcome; observational diet–cognition associations
Full peer-reviewed report in English, any year	Conference abstracts, protocols, registry entries and non-English reports

Abbreviations: MCT, medium-chain triglyceride; PD, Parkinson’s disease; PD-MCI, Parkinson’s disease mild cognitive impairment; PDD, Parkinson’s disease dementia.

**Table 2:** Design and population of the 23 controlled studies.

Study	Design	Randomized/Analyzed for Cognition	PD-MCI an Enrollment Criterion	Intervention vs. Comparator	Duration	Adherence or Exposure Verification
Paknahad 2020 [53]	Parallel RCT, single center	80/70	No (idiopathic PD)	Individualized Mediterranean diet vs. healthy-diet advice	10 weeks	24-h dietary recalls every 2 weeks, weekly telephone follow-up and monthly counseling; 5 excluded for non-adherence
Krikorian 2019 [54]	Randomized pilot	18/14	Yes	Low-carbohydrate vs. high-carbohydrate diet	8 weeks	Weekly diet diaries and interim ketone checks; βHB rose to 0.31 ± 0.19 mM
Phillips 2018 [55]	Pilot RCT	47/47 ITT; 38 completed	No	Ketogenic vs. low-fat diet	8 weeks	Daily blood ketones; mean 1.15 vs. 0.16 mmol/L
Choi 2024 [56]	Double-blind feasibility RCT, inpatient	16/16 randomized week; 15 at week 3	No	MCT-supplemented ketogenic vs. standard diet	1 week (plus 2-week open-label extension)	Controlled inpatient feeding; ketosis measured
Tosefsky 2026, KIM [50]	Random-order crossover, two active arms	52 (48 started)/33 completed both phases	No (MoCA ≥ 21 required)	Mediterranean–ketogenic vs. Mediterranean plus MCT	8 weeks per phase, 8-week washout	Blood ketones measured; biochemical ketosis in about half
Hiller 2018 [57]	Double-blind placebo-controlled pilot RCT	58/51 completers; MoCA *n* = 50	No	Vitamin D ≈ 10,000 IU/day vs. placebo (calcium in both)	16 weeks	Serum 25-hydroxyvitamin D rise documented
NET-PD LS-1 2015 [49]	Multicenter double-blind placebo-controlled RCT	1741/955 at 5-year interim analysis	No (early treated PD)	Creatine 10 g/day vs. placebo	Median 4 years	Study-drug exposure 71% vs. 79% of participant-years; 34% vs. 26% stopped study drug
QE3 2014 [58]	Multicenter double-blind placebo-controlled RCT	600/ITT	No (early PD; cognitively impaired excluded)	CoQ10 1200 or 2400 mg/day vs. placebo (vitamin E in all)	Mean 10.4 months	Medication logs and plasma CoQ10 at baseline and months 1/8/16; compliance reported similar between groups
Li 2015 [59]	Randomized placebo-controlled, single center	75/75 (tables retain 38 and 37)	Yes	Creatine 5 g twice daily plus CoQ10 100 mg three times daily vs. placebo	18 months	Not reported in the full article
Lam 2026 [60]	Double-blind placebo-controlled RCT	61/61 (ITT; 51 completed)	No (PD with anxiety; more impaired excluded)	Nine-strain probiotic vs. placebo	12 weeks	Returned-sachet counts; mean 98.5% in both groups
Yulug 2025 [61]	Double-blind placebo-controlled phase II RCT (2:1)	48/43	No	Combined metabolic activators vs. placebo	84 days	Protocol: weekly nurse telephone checks of compliance and adverse events; achieved adherence percentage not reported
Hajhashemy 2026 [62]	Triple-blind placebo-controlled parallel RCT	92/92 ITT with EM imputation; 84 completed (43/41)	No (AMT 8.1 of 10 at baseline in both arms)	Saffron tablets 100 mg/day (20.4 mg crocin) vs. lactose placebo	12 weeks	Telephone checks and tablet counts; achieved adherence percentage NR
Anamnart 2026 [63]	Randomized, no placebo (open), plus 60 healthy comparators	60/60 PD participants (30/30)	No	Vitamin D3 2000 IU/day vs. no additional treatment	12 weeks	Self-reported adherence; serum 25(OH)D rise documented
Wakade 2021 [39] ^†^	Double-blind placebo-controlled RCT, single center	47 enrolled/39 evaluated at 6 months (18 niacin, 21 placebo); ITT and per-protocol reported	No (MMSE >24; dementia excluded)	Niacin 250 mg/day vs. placebo	6 months (then open-label niacin)	Quantitative adherence NR
Sun 2022 [40] ^†^	Double-blind placebo-controlled RCT	100 (50/50)/clinical set 82 (48 probiotic, 34 placebo); omics set 74 (45/29)	No	Bifidobacterium animalis subsp. lactis Probio-M8 vs. placebo, added to benserazide and dopamine agonists	3 months	Strain engraftment documented; quantitative adherence NR
Huang 2023 [44] ^†^	Randomized two-arm trial; random-number table	80 (40/40)/outcome-specific n NR	No	Ginkgo leaf tablets plus compound carbidopa vs. compound carbidopa alone	3 months	NR
Shateri 2025 [45] ^†^	Double-blind placebo-controlled RCT	58/12 and 15 (six-month MoCA)	No (MoCA 17–30 at entry; baseline MoCA 22.4 ± 4.9 vs. 21.0 ± 6.9)	Boswellia serrata extract standardized to boswellic acids, 1200 mg/day, vs. microcrystalline-cellulose placebo	6 months	Weekly pill counts, self-report and caregiver logs; per-protocol exclusion if >15% of doses missed; compliance figures NR
Suzuki 2013 [38] ^†^	Double-blind placebo-controlled RCT, single center	114/authors’ ITT 112 (55/57); MMSE-specific denominator qualified in Table S1	No (severe dementia excluded)	Vitamin D3 1200 IU/day vs. placebo	12 months	Bottle checks at visits; compliance 89% vs. 97%; serum 25-hydroxyvitamin D rise documented
DATATOP 1993/1994 [36,37] ^†^	Multicenter double-blind placebo-controlled 2 × 2 factorial RCT	800 randomized; 799 baseline cognitive assessments; 710 longitudinal (174/174/187/175: placebo/tocopherol/deprenyl/both)	No (early untreated PD; MMSE ≥23; mean baseline MMSE 28.8)	Tocopherol 2000 IU/day vs. placebo (factorial with deprenyl)	Mean 14 ± 6 months; cognitive visits every 6 months	97.9–99.5% of dispensed doses taken; serum tocopherol consistent with assignment in 78–90% (primary report)
Zeng 2024 [41] ^†^	Double-blind placebo-controlled RCT (1:1)	46/ITT 46 (23/23); per-protocol 42 (21/21)	No; severe cognitive impairment excluded	Bacteroides fragilis BF839 plus earthworm protein vs. placebo	12 weeks	<20% of required dose was an exit criterion; quantitative adherence NR
Magistrelli 2024 [42] ^†^	Randomized placebo-controlled trial; blinding stated, roles unspecified	40 enrolled; 38 completed; one dropout supplied clinical data; exact MoCA n NR	No	Three-strain Bifidobacterium formulation with fructo-oligosaccharides vs. maltodextrin	12 weeks	NR; product supplied by manufacturer
Bolner 2023 [43] ^†^	Randomized double-blind placebo-controlled crossover, single center	39/NR	No (Hoehn and Yahr 1–2)	Fermented papaya preparation 9 g/day vs. placebo, 180 days per period; no washout reported	180 days per period (360 days total)	NR; manufacturer and distributor support
Chong 2021 [51] ^†^	Double-blind, three-arm randomized phase; later open-label phase excluded from controlled evidence	47 randomized (16/16/15); 46 completed 3 months (16/15/15); cognitive-specific analyzed n NR; allocation from Figure 1 in Chong et al. [51]	No explicit PD-MCI criterion; baseline MMSE near ceiling	Slow-release niacin 100 mg/day or 250 mg/day vs. placebo	3 months (randomized phase only)	Quantitative adherence NR; later biomarker findings do not establish randomized-phase adherence

^†^ Identified by the structured search run for this revision (Section 2.2). Abbreviations: CoQ10, coenzyme Q10; ITT, intention-to-treat; EM, expectation–maximization; KIM, Mediterranean–Ketogenic Interventions in Parkinson’s disease trial; MCT, medium-chain triglyceride; MMSE, Mini-Mental State Examination; MoCA, Montreal Cognitive Assessment; NR, not reported; PD, Parkinson’s disease; PD-MCI, Parkinson’s disease mild cognitive impairment; RCT, randomized controlled trial; AMT, Abbreviated Mental Test.

**Table 3:** Cognitive outcomes, between-group estimates and appraisal of the 23 controlled studies.

Study	Cognitive Instrument; Endpoint Status	Prespecified; Multiplicity	Between-Group Result (95% CI)	Classification	Attrition	Main Appraisal Concerns
Paknahad 2020 [53]	Persian MoCA; study objective (primary)	Yes; six subsections, no correction reported	Total MoCA *p* = 0.001; CI not reported; calculated difference in change 2.22 points (unadjusted)	Favorable objective between-group difference (primary)	12.5%; completer analysis	Post-randomization exclusion of non-adherent participants; dietary masking unclear; short duration
Krikorian 2019 [54]	Lexical access and memory composites; primary	Yes; several measures, no correction reported	*p* = 0.02 (f = 0.76) and *p* = 0.01 (f = 0.87); CI not reported	Favorable objective between-group difference (primary, pilot)	22%; completer analysis	14 analyzed; diets unblinded; concurrent weight and metabolic change
Phillips 2018 [55]	MDS-UPDRS Part I cognitive-impairment item; exploratory	No (item-level); multiple non-motor items	Cognitive-item change difference −0.27 points; item-specific CI/p not given in main text	Subjective only	9/47 (19%); ITT with imputation	Single patient-rated item; no objective battery; diets unblinded
Choi 2024 [56]	3-back, Stroop, reaction time; exploratory	No; multiple measures	Similar improvement in both groups; arm scores in Table S5; comparative estimates/CIs NR	No between-group difference (exploratory)	0/16 randomized week; 1/16 in extension	16 participants; one-week comparison; feasibility design
Tosefsky 2026, KIM [50]	MoCA and PD-CRS; exploratory	Yes (secondary); ITT and available-case analyses	No MoCA difference; PD-CRS 2-point decrease in available cases only, not ITT	No between-group difference (exploratory)	37%	High dropout; ketosis in about half; diets unblinded
Hiller 2018 [57]	MoCA and neuropsychological battery; secondary	Yes; several tests	MoCA change +0.9615 vs. +1.042 (*p* = 0.905); other tests in Table S4	No between-group difference (secondary)	12%; completer analysis	Powered for balance, not cognition
NET-PD LS-1 2015 [49]	SDMT within global primary outcome; SCOPA-Cog secondary	Yes; single global outcome	SDMT worsening contrast −0.4 (95% CI −2.7 to 1.8), placebo minus creatine; SCOPA-Cog null	No between-group difference (component of primary)	668/874 and 669/867 retained at interim cutoff; 955 eligible for 5-year analysis	Limited cognitive-domain coverage; early PD
QE3 2014 [58]	Symbol Digit Modalities Test; secondary	Yes	Adjusted SDMT changes −0.49/−3.36 vs. −3.02; no significant difference; stopped for futility	No between-group difference (secondary)	65/600 (10.8%) premature withdrawals	Early PD; cognitively impaired excluded
Li 2015 [59]	MoCA; primary	Yes; single instrument at 12 and 18 months	*p* < 0.01 at 18 months; CI not reported; calculated endpoint difference 5.22 points (unadjusted); both groups declined	Favorable objective between-group difference (primary); unreplicated	NR; flow not described	Blinded roles incompletely specified; missing-data handling NR; single center; components not separable
Lam 2026 [60]	MoCA; secondary (primary outcome anxiety, negative)	Yes; multiple secondary outcomes, no correction reported	Adjusted mean difference +1.1 points (0.04 to 2.1); *p* = 0.043	Favorable objective between-group difference (secondary)	16%; ITT	Lower CI limit near zero; multiplicity; more impaired participants excluded
Yulug 2025 [61]	MoCA; secondary	Yes; multi-omics and subgroup analyses	Between-group MoCA *p* = 0.233/0.219/0.564 at days 0/28/84; arm scores in Table S2	No significant between-group difference at the reported time points; within-arm improvement does not establish benefit.	10%	Baseline imbalance; subgroup and molecular inference
Hajhashemy 2026 [62]	AMT (10-item screen); secondary	Article-defined secondary; multiple outcomes, no correction reported	Baseline-adjusted difference +0.78 points (95% CI 0.16 to 1.40), *p* = 0.01	Favorable objective between-group difference (secondary)	8/92 (8.7%): 3 saffron, 5 placebo; ITT *n* = 92 with EM imputation	Brief screening instrument near ceiling; small absolute change; many outcomes without correction; no replication
Anamnart 2026 [63]	Thai MMSE; primary clinical outcome	Yes	Difference in Thai MMSE change +0.43 (95% CI −0.54 to 1.41; *p* = 0.378)	No between-group difference (primary)	0/60; all PD participants analyzed	No placebo; no blinding; brief follow-up
Wakade 2021 [39]	Trail Making Test B − A (cognitive flexibility), Stroop; secondary (primary outcome UPDRS III)	Article describes secondary outcomes; registry prespecification not independently checked; Bonferroni adjustment stated	Reported comparative estimates: TMT B − A − 6.04 s (95% CI −54.93 to 42.85); Stroop 1.96 (−4.16 to 8.08)	No between-group difference (secondary)	8/47 (17%) before 6 months; cognitive missing-data handling not separately explicit	Powered for motor outcome; small; mostly male veterans
Sun 2022 [40]	MMSE; clinical index, formal hierarchy NR	Listed in Methods; registry prespecification not independently checked; no MMSE multiplicity correction reported	Between-group MMSE *p* = 0.71 at baseline, 0.57 at 1 month, 0.44 at 3 months; change-based contrast and CI NR	No between-group difference on reported comparisons	18/100: 2/50 vs. 16/50 during intervention	Differential exclusions; clinical *n* = 82, not omics *n* = 74; cross-sectional tests; multiple outcomes
Huang 2023 [44]	MMSE and MoCA; study objectives, formal hierarchy NR	Prospective prespecification not established; no adjustment reported	Post-treatment MMSE and MoCA higher in combination group (*p* < 0.05); estimates and CI not reported	Favorable objective between-group difference; hierarchy NR; limited methodological reporting	NR	Random-number table reported; concealment, blinding, attrition and numeric cognitive estimates NR
Shateri 2025 [45]	MoCA at baseline and 6 months; secondary (primary clinical outcome UPDRS III; registered primary outcome cytokines)	MoCA not a registered outcome; UPDRS, MoCA and 12 cytokines without adjustment	Within-group: 22.42 ± 4.93 → 23.67 ± 3.82 (*p* = 0.19) with boswellic acids; 21.00 ± 6.92 → 21.40 ± 7.33 (*p* = 0.86) with placebo; between-group contrast NR	Within-group results only; comparative effect not established	14 of 58 (24%) withdrew; six-month MoCA in 12 of 29 vs. 15 of 29	Within-group comparisons only; six-month MoCA in 27 of 58; baseline values differ between the report’s tables; three deaths among withdrawals; manufacturer-affiliated author
Suzuki 2013 [38]	Japanese MMSE; article-defined primary measure	Registry prespecification not independently checked; permutation procedures reported	Change −0.33 ± 2.16 vs. +0.27 ± 1.74, *p* = 0.12; not worsened 63.3% vs. 78.2%, risk difference −0.15 (−0.32 to 0.02)	No between-group difference (primary)	10/114 (8.8%); authors’ ITT 112; MMSE denominator qualified in Table S1	Nominal direction favored placebo; MMSE ceiling (median 28); powered for Hoehn and Yahr outcome
DATATOP 1993/1994 [36,37]	Digit Span, Symbol Digit, Selective Reminding, Odd Man Out, Spot-the-Dot, verbal fluency and MMSE; cognition secondary	Prospective cognitive assessments; Bonferroni *p* < 0.0033 for 15 variables	No significant tocopherol effect after correction; adjusted estimate and 95% CI not reported; arm-level scores in Table S3	No between-group difference (secondary)	90/800 (11.3%) excluded before 6 months because of endpoint or early termination; 710 in cognitive table	Early untreated PD; MMSE ≥23; little cognitive decline; cognitive analysis excluded early endpoints/terminations and was not ITT; pre-registration era
Zeng 2024 [41]	MMSE; formal primary/secondary designation NR	Listed in Methods; registry prespecification not independently checked; adjustment NR	ITT MMSE Δ12 weeks +0.39 ± 1.58 vs. +0.57 ± 1.40; between-group *p* = 0.650 (6 weeks *p* = 0.335); CI NR	No between-group difference; hierarchy NR	4/46 (8.7%); ITT 46 and per-protocol 42	Small; baseline MMSE near ceiling; multiple outcomes; imputation method NR
Magistrelli 2024 [42]	MoCA; formal hierarchy NR	Prospective prespecification not established; multiple outcomes, adjustment NR	Within-group: 27.47 → 28.05 (*p* = 0.078) with probiotic, 26.30 → 25.70 (*p* = 0.065) with placebo; between-group contrast NR	Within-group results only; comparative effect not established	2/40 (5%) dropped out; one supplied clinical data; exact MoCA n NR	Within-group comparisons only; MoCA near ceiling; manufacturer-supplied product; preliminary data
Bolner 2023 [43]	MoCA and FAB; formal hierarchy NR	Prospective prespecification not established; multiple tests without reported adjustment	Pooled periods: MoCA within-period *p* = 0.015 (active) vs. 0.734 (placebo); FAB nonsignificant in both; between-treatment contrast and score means NR	Within-group results only; comparative effect not established	NR	No washout reported; no period/carryover model; pooled periods; analyzed n NR; industry support
Chong 2021 [51]	Trail Making Test B, first trial; nonprimary (UPDRS III primary); prospective cognitive hierarchy not verified	Cognitive tests listed in Methods; three-month prespecification and multiplicity procedure NR	Baseline → 3 months, mean ± SD (s): placebo 73.6 ± 30.5 → 69.7 ± 44.9; 100 mg 109.3 ± 138.6 → 104.7 ± 123.5; 250 mg 95.3 ± 70.8 → 92.5 ± 72.8. Comparative test/CI NR	Descriptive arm-level data only; no between-group cognitive inference	1/47 (100-mg arm) dropped out before 3 months; reason and cognitive-specific n NR	Small trial; large baseline dispersion; no comparative cognitive estimate; later uncontrolled phase not efficacy evidence

Classification follows the rule in Section 2.5; Sun 2022 [40] is classified as no between-group difference because all reported MMSE comparisons were nonsignificant; its clinical and omics analysis populations are distinguished. Endpoint hierarchy is marked NR when not established from the report, rather than inferred from a study objective. Abbreviations: AMT, Abbreviated Mental Test; CI, confidence interval; ITT, intention-to-treat; EM, expectation–maximization; KIM, Mediterranean–Ketogenic Interventions in Parkinson’s disease trial; MDS-UPDRS, Movement Disorder Society Unified Parkinson’s Disease Rating Scale; MMSE, Mini-Mental State Examination; MoCA, Montreal Cognitive Assessment; NR, not reported; PD, Parkinson’s disease; PD-CRS, Parkinson’s Disease–Cognitive Rating Scale; UPDRS, Unified Parkinson’s Disease Rating Scale; FAB, Frontal Assessment Battery; SD, standard deviation; SDMT, Symbol Digit Modalities Test; TMT, Trail Making Test; SCOPA-Cog, Scales for Outcomes in Parkinson’s Disease–Cognition. Study designs and populations are detailed in Table 2.

### 2.4. Study Selection and Data Extraction

Najeeb Qadi performed retrieval, screening, eligibility decisions and data extraction. Amaal Aldakheel verified every entry in Table 2, Table 3, Tables S1 and S2 against source reports; disagreements were resolved by discussion. All 23 included studies were assessed from full reports, including the ten revision-stage additions. The dispositions and retrieval bases of all 33 selected reports were checked against the screening log. No independent duplicate screening was performed, and no unpublished data were requested. Extracted items included study design; enrollment, randomization, completion and analysis populations; PD-MCI eligibility; intervention, comparator and duration; adherence or exposure verification; cognitive instruments, endpoint hierarchy and prespecification; between-group estimates and 95% confidence intervals (CIs); attrition; and adverse events. Published estimates were retained, unavailable CIs were marked as not reported, and effect sizes were not imputed from significance levels. Two contrasts calculated from published means (Paknahad and Li) were labelled as unadjusted descriptive values, not adjusted treatment effects. For Yulug et al., visit-specific MoCA means and sample SDs were calculated from Supplementary Dataset 1 accompanying their published article [61], using available observations without imputation; published inferential tests were retained. Trial-specific extraction details are provided in Section 5 and the Supplementary Tables.

### 2.5. Critical Appraisal and Outcome Classification

Studies were classified as follows: favorable objective between-group difference, when a reported objective cognitive comparison significantly favored the intervention; no between-group difference, when the comparison was nonsignificant; within-group results only, when within-arm analyses were reported without a between-group cognitive contrast, regardless of significance; descriptive arm-level data only, when cognitive summaries lacked a comparative test or estimate; or subjective only, when cognition was assessed solely by a patient- or clinician-rated symptom item. Endpoint status (primary, secondary, exploratory or not established) was recorded to distinguish evidential weight.

Appraisal used domains of the revised Cochrane risk-of-bias tool for randomized trials (RoB 2) [64], covering randomization, allocation concealment, participant and assessor blinding, missing data, selective reporting and prespecification, alongside multiplicity, adherence and analysis population. Supplementary Table S1 records domain-level concerns and unavailable information. Given the narrative design and differing comparator and crossover structures, no overall RoB 2 rating, summary score or GRADE classification was assigned.

### 2.6. Synthesis

The unit of evidence is the trial, not each subtest or publication. No pooled estimate, meta-analysis or statistical assessment of publication bias was undertaken. Findings are organized by the three therapeutic claims (nutritional rescue, short-term cognitive performance and progression) and by endpoint hierarchy. A favorable within-group change is not treated as a treatment effect. Null results are interpreted in relation to precision and endpoint sensitivity, not as proof of equivalence. Where a between-group difference on a screening instrument is reported, its size is compared with the practice effects and reliable-change thresholds available for such instruments (Section 6.2) because a statistically significant screening difference is not the same as improved cognition.

## 3. Mechanistic Pathways Linking Nutrition to Cognition in Parkinson’s Disease

This section summarizes four pathways through which nutrition could plausibly affect cognition in PD: gut–brain and immune signaling, one-carbon metabolism, mitochondrial energetics and nutritional reserve, and the cognitive phenotype that an intervention targets. Each pathway is a hypothesis that motivates trials; none has been shown to mediate a cognitive effect in people with PD.

### 3.1. Gut–Brain Signaling, Microbial Metabolites and Neuroinflammation

The gut microbiome of people with PD differs from that of controls. A pooled re-analysis of ten 16S rRNA datasets found consistent enrichment of Lactobacillus, Akkermansia and Bifidobacterium and depletion of Lachnospiraceae and Faecalibacterium, which are major producers of short-chain fatty acids (SCFAs) [65], and a large metagenomic study found that more than 30% of species, genes and pathways tested were altered in PD [66]. These differences are modest in size and vary with antiparkinsonian medication, constipation, sequencing method, geography and diet [12,65,67]. Fecal SCFA concentrations are reduced in PD [68], and reduced SCFAs are accompanied by markers of intestinal inflammation and barrier dysfunction, which are not reflected in plasma inflammatory profiles [69]. SCFAs regulate epithelial integrity, mucosal immunity and systemic inflammatory tone [70], and translational work links intestinal barrier dysfunction and Toll-like receptor 4 signaling to the gut–brain axis in PD [71]. In one PD cohort, plasma and fecal SCFA patterns were related to clinical severity and cognition in compartment-specific ways [72].

Neuroinflammation and systemic immune activation are associated with PD progression and with dementia risk [73,74]. Mediterranean-style patterns reduce cardiovascular events and improve cardiometabolic risk profiles in the general population [75], which offers a plausible route to brain health. The key distinction is between association and mediation. Microbiome composition, metabolite levels and inflammatory markers are associated with PD and with cognition, but no PD trial has shown that a diet-induced change in any of these markers mediates a durable cognitive effect. Neither recent trial establishes that changes in the microbiome or inflammatory markers mediate a cognitive benefit [50,60]. Mechanistic outcomes should therefore be presented as explanatory hypotheses rather than as surrogate proof of neuroprotection.

### 3.2. One-Carbon Metabolism

One-carbon metabolism is the network of folate- and vitamin B12-dependent reactions that transfers single-carbon units for methylation and nucleotide synthesis [76]. Levodopa is O-methylated by catechol-O-methyltransferase, which consumes S-adenosylmethionine and raises homocysteine, particularly when folate or vitamin B12 status is marginal [77,78]. Observational studies associate higher homocysteine with worse cognitive performance in PD, and a meta-analysis found higher homocysteine and lower folate and vitamin B12 in cognitively impaired than in cognitively normal PD groups [28,79]. Residual confounding, reverse causation, renal function, nutritional status and levodopa exposure remain important. The pathway is clinically actionable when deficiency is present, but lowering homocysteine has not been shown to prevent PD-MCI or PDD.

### 3.3. Mitochondrial Energetics, Vitamin D and Nutritional Reserve

Ketone bodies provide an alternative oxidative substrate for the brain and modulate inflammatory signaling [80]; CoQ10 and creatine have mechanistic links to mitochondrial energy handling. These rationales motivated human trials, but the large negative monotherapy trials described in Section 5.4 show the limited predictive value of preclinical plausibility. Vitamin D has neurotrophic and immunomodulatory roles [81], and cross-sectional PD data link higher 25-hydroxyvitamin D with better verbal fluency and memory [29]; intervention data are negative for cognition (Section 5.2).

Nutritional reserve is the capacity of energy stores, lean mass and micronutrient status to buffer the metabolic demands of illness. It is a distinct and clinically important issue in PD. Malnutrition or risk of malnutrition is common [30]; oropharyngeal dysphagia affects a substantial proportion of patients [31], and early weight loss predicts faster subsequent decline in global and executive cognition, although this association does not show that intentional weight gain prevents decline [32]. Restrictive interventions, including ketogenic regimens and chronic protein restriction, can reduce energy intake, lean mass and micronutrient intake and must be judged against the competing risks of malnutrition and sarcopenia, not only against their theoretical neurobiological benefit [33,82]. The converse is also evidence-based: in a randomized trial of 150 cognitively intact inpatients with parkinsonism undergoing intensive rehabilitation, a whey protein supplement enriched with leucine and vitamin D improved walking distance and preserved muscle mass, with no cognitive endpoint [83], and a 12-week randomized trial of individualized dietetic advice in 19 malnourished community-dwelling people with PD improved nutritional status without a between-group difference in quality of life [84].

### 3.4. Preclinical Evidence

Animal and cellular studies support biological plausibility but do not predict clinical efficacy. In the 1-methyl-4-phenyl-1,2,3,6-tetrahydropyridine (MPTP) mouse model, D-β-hydroxybutyrate infusion rescued mitochondrial respiration and reduced dopaminergic neuron loss [85], and a ketogenic diet reduced motor dysfunction, neuronal loss and inflammation while reshaping the gut microbiota and metabolome [86]. In an α-synuclein-overexpressing mouse model, gut microbes were required for motor deficits and microglial activation, and transplantation of microbiota from people with PD worsened the phenotype [87]. The large human trials of creatine and CoQ10 did not demonstrate clinical benefit despite their preclinical rationale [49,58]. Preclinical results are therefore treated here as hypothesis-generating; they define candidate mechanisms and biomarkers but not the population, dose or endpoint of a cognitive trial.

### 3.5. Cognitive Phenotype and the Target of Intervention

PD-MCI is defined by cognitive decline and objective impairment with largely preserved functional independence; PDD additionally requires deficits that interfere with everyday function beyond the contribution of motor or autonomic disability [9,10]. Attention and executive dysfunction may coexist with visuospatial, language or memory impairment. A trial enrolling broadly defined PD therefore mixes people with different baseline deficits, ceiling effects and risks of progression, and a change in a global score can conceal improvement in one domain and deterioration in another.

The mechanistic hypothesis should determine the population and endpoint. A deficiency-correction trial should document the deficiency and its correction. A ketogenic study should establish exposure to ketosis and select outcomes appropriate to the enrolled cognitive phenotype. A trial intended to preserve nutritional reserve should measure intake, weight trajectory and function alongside cognition. None of these designs can presume that a biomarker identifies a responsive subgroup without a prospectively specified interaction analysis.

In summary, the mechanistic literature justifies testing nutrition against cognitive endpoints in PD, but every pathway described here is at the stage of association or animal evidence. The controlled human studies that follow should be read with that starting point in mind.

## 4. Dietary Patterns: Controlled Evidence and Appraisal

Five controlled studies tested a dietary pattern with a cognitive outcome in established PD: one Mediterranean-diet trial and four ketogenic or MCT-supplemented interventions. Protein redistribution has no controlled cognitive evidence and is discussed for its practical relevance. Design and population details are given in Table 2, cognitive outcomes and appraisal in Table 3, and baseline-to-follow-up scores, adherence, attrition and adverse events in Supplementary Table S2.

### 4.1. Mediterranean and MIND Patterns

Observational studies associate Mediterranean or MIND adherence with lower incident parkinsonism, fewer prodromal features or later PD onset [16,17,18]. A meta-analysis of 12 observational studies registered in the International Prospective Register of Systematic Reviews (PROSPERO) reported a pooled odds ratio of 0.75 (95% CI 0.66–0.84) for PD in the highest versus lowest Mediterranean-adherence category, with a stronger association for prodromal than for diagnosed PD [19]. These studies provide etiological context, not evidence that the same diet improves cognition after PD is established.

Paknahad et al. randomized 80 participants with idiopathic PD to an individualized Mediterranean diet or healthy-diet advice for 10 weeks, with Persian Montreal Cognitive Assessment (MoCA) as the study objective [53]. Five intervention participants were excluded for non-adherence, and five controls withdrew, leaving 35 per group in a completer analysis. The reported between-group *p* value for total MoCA was 0.001 (general linear model), with significant differences for executive function, language and attention subsections and none for visuospatial ability, recall or orientation; no between-group CI was published. A difference in change of 2.22 MoCA points can be calculated from the published means (+1.61 versus −0.61); this is an unadjusted descriptive value calculated by the review authors, not a prespecified adjusted treatment effect (Supplementary Table S2). Excluding participants on the basis of post-randomization adherence breaks the protection afforded by randomization and can exaggerate efficacy because adherent participants may differ from non-adherent ones in health, support and motivation. Participant and assessor blinding were reported, but how participants could be masked during individualized dietary counseling is unclear, and uncertain equivalence of counseling contact, multiple subsection comparisons and the short follow-up further limit interpretation. The trial does not establish durable functional benefit or dementia prevention.

No completed randomized trial of the MIND diet as a treatment for PD-MCI was identified. Outside PD, a three-year randomized trial involving 604 older adults without cognitive impairment found that the MIND diet did not outperform a control diet with mild caloric restriction on global cognition or MRI outcomes [88]. This is not direct evidence against MIND in PD-MCI, but it shows that observational associations and a cognition-oriented diet name are insufficient grounds for a treatment claim. A Mediterranean-style pattern remains a reasonable default because of overall dietary quality and cardiovascular benefit [75]; the cognition-specific claim in PD rests on one short, single-center trial.

### 4.2. Ketogenic and Mediterranean–Ketogenic Interventions

Nutritional ketosis is a metabolic state, usually defined by blood β-hydroxybutyrate of at least 0.5 mmol/L, and prescribing a ketogenic diet does not guarantee it. Severe carbohydrate restriction, an MCT supplement added to a Mediterranean pattern and a weight-reducing low-carbohydrate diet differ in ketone production, fiber, fatty-acid composition, energy intake and behavioral burden [50,54,55,56]. Whether each study verified exposure to ketosis is therefore recorded in Table 2 rather than discussed only narratively.

Krikorian et al. enrolled 18 participants with PD-MCI in an eight-week randomized pilot of a low- versus high-carbohydrate diet; 14 completed, seven per group (22% attrition) [54]. Baseline-adjusted comparisons favored the low-carbohydrate group for lexical access (*p* = 0.02; Cohen’s f = 0.76) and memory composites (*p* = 0.01; f = 0.87); memory interference did not reach significance (*p* = 0.06), and CIs were not reported. These are large standardized effects from a 14-person completer analysis in a predominantly male sample with concurrent metabolic and weight changes. The result supports a short-term performance hypothesis with substantial uncertainty.

Phillips et al. randomized 47 participants to ketogenic or low-fat diets for eight weeks; 38 completed [55]. Both groups improved, with a larger reduction in Movement Disorder Society Unified Parkinson’s Disease Rating Scale (MDS-UPDRS) Part I in the ketogenic group and a difference in its patient-rated cognitive-impairment item. No objective cognitive battery was used, so this study contributes a subjective result only. Choi et al. randomized 16 participants to an MCT-supplemented ketogenic diet or a standard diet during a one-week inpatient phase; all 16 completed that randomized phase. Fifteen completed the subsequent two-week open-label extension. Exploratory three-back and Stroop measures showed no between-group cognitive difference [56].

The Mediterranean–Ketogenic Interventions in Parkinson’s Disease (KIM) trial is the most informative recent study [50]. Fifty-two participants without dementia were randomized to two eight-week Mediterranean–ketogenic interventions in random order with an eight-week washout; 48 started and 33 completed both phases (37% dropout). Neither total MoCA nor the Parkinson’s Disease–Cognitive Rating Scale (PD-CRS) improved; a two-point PD-CRS decrease after the Mediterranean–ketogenic phase appeared only in the available-case analysis and not in the intention-to-treat (ITT) analysis. Biochemical ketosis was documented in only about half of participants during the carbohydrate-restricted phase. The negative cognitive findings therefore apply to the dietary programs as implemented, not to sustained ketosis itself; equally, restricting analysis to participants who achieved ketosis would select for adherence and weaken randomization. The two 2026 reviews reach the same conclusion: the clinical ketogenic literature in PD consists of small studies with heterogeneous protocols, variable ketone targets and high dropout [23,24]. One of them tabulates a six-month Chinese-language randomized controlled trial (RCT) of adjunctive ketogenic diet with motor and activities-of-daily-living outcomes [24]; that report was outside the English-language boundary of this review and did not assess cognition.

A 2026 RCT of a personalized, plant-rich, low-glycemic dietary intervention with time-restricted eating in 40 participants with PD reported improvement in the primary motor endpoint and in apathy, activities of daily living and bowel function over six months [89]. It included no cognitive outcome and is therefore contextual, but it is relevant to future design: it combined several metabolic principles, individualized the prescription and retained participants for six months. Ketogenic and related diets should remain experimental for cognition in PD and should be supervised by a dietitian and a physician in research settings.

### 4.3. Protein Redistribution and Levodopa Response

Dietary large neutral amino acids compete with levodopa for intestinal absorption and transport across the blood–brain barrier. Protein redistribution, defined as preserving adequate daily protein while shifting most of it to the evening, can improve motor fluctuations in selected levodopa-treated patients, although the literature is heterogeneous, and long-term adherence is difficult [90,91]. A large observational study linked protein amount and distribution with levodopa requirements and motor complications [92]. Neither source establishes a cognitive effect. Better motor “on” time may improve apparent test performance or daily function without modifying neurodegeneration, and chronic protein restriction can worsen weight loss and sarcopenia. Protein timing should be individualized for reproducible dose–meal interactions, supervised by a dietitian where possible, and separated from claims about cognitive preservation.

In summary, dietary-pattern evidence for cognition in PD consists of one positive 10-week Mediterranean trial analyzed using a completer analysis, one positive 14-person ketogenic pilot, one subjective ketogenic result and two ketogenic studies with no between-group cognitive difference, including the largest and longest. No dietary pattern has replicated objective cognitive benefit, and none has been tested against progression to PDD.

## 5. Micronutrients and Supplements: Controlled Evidence and Appraisal

Eighteen randomized controlled studies tested a supplement with a cognitive outcome: three vitamin D trials, two niacin trials, the DATATOP tocopherol comparison, two large creatine and CoQ10 monotherapy trials, one creatine–CoQ10 combination trial, four probiotic trials, one combined metabolic activator trial and four botanical or functional-food trials (saffron, boswellic acids, ginkgo leaf tablets and fermented papaya). Ten of the 18 were identified by the structured search run for this revision. Vitamin B12, folate and omega-3 fatty acids have controlled biochemical or motor evidence but no controlled cognitive outcome and are discussed as context.

### 5.1. Folate, Vitamin B12, Niacin and Homocysteine

Vitamin B12 and folate supplementation lower homocysteine in levodopa-treated PD. A randomized study compared vitamin B12 with folate against entacapone for their effect on homocysteine; the biochemical endpoint improved, but cognition was not a treatment endpoint [77]. The Romanian open-label pilot combined vitamin B12, folic acid and vitamin D3 for six months and reported lower homocysteine with improved health-related quality-of-life domains [52]; the PDQ-39 cognition domain is patient-reported and the study had no control group. An analysis of 680 baseline samples from early untreated PD in the DATATOP cohort found that lower vitamin B12 predicted greater mobility deterioration, whereas elevated homocysteine predicted greater decline on the Mini-Mental State Examination (MMSE) [78]. Because exposure was measured before levodopa treatment, the association cannot be attributed solely to levodopa methylation; vitamin status was not randomized, however, and the study did not test supplementation. The defensible clinical conclusion is to identify and correct documented vitamin B12 or folate deficiency, with homocysteine or methylmalonic acid measured when the clinical context warrants, and not to prescribe high-dose B vitamins to replete patients in order to prevent cognitive decline.

Niacin (vitamin B3) is the one B vitamin with a randomized cognitive outcome in PD. Wakade et al. randomized 47 participants with PD to niacin 250 mg/day or placebo for six months in a double-blind, registered trial powered for the motor primary outcome; 39 completed the blinded phase (21 placebo, 18 niacin), and an open-label niacin phase followed [39]. Cognition was secondary: the reported comparative estimate at six months was −6.04 s (95% CI −54.93 to 42.85) for cognitive flexibility (Trail Making Test B − A) and 1.96 (95% CI −4.16 to 8.08) for the Stroop test, with Bonferroni adjustment for multiplicity; neither differed, and dementia was an exclusion criterion. Chong et al. reported an earlier three-month randomized, double-blind phase comparing placebo, niacin 100 mg/day and niacin 250 mg/day, followed by uncontrolled open-label supplementation [51]. Figure 1 in Chong et al. [51] documented 47 randomized participants: 16 placebo, 16 niacin 100 mg and 15 niacin 250 mg. One participant in the 100 mg arm dropped out, leaving 46 completers (16/15/15). These allocation and completion counts are used here; the Methods text gives 15 for the 100 mg arm at allocation. Table 2 in Chong et al. [51] gives Trail Making B means ± SD at baseline and three months: placebo 73.6 ± 30.5 to 69.7 ± 44.9 s; 100 mg 109.3 ± 138.6 to 104.7 ± 123.5 s; and 250 mg 95.3 ± 70.8 to 92.5 ± 72.8 s. No three-month between-arm cognitive test or CI is reported, so these are descriptive data rather than evidence of a favorable or null comparative effect. Only the randomized phase enters the controlled evidence set; later uncontrolled findings are contextual. Wakade describes its six-month two-arm protocol separately from this earlier three-arm trial, although individual participant overlap is not explicitly addressed [39]. A randomized comparison of folate plus vitamin B12 against entacapone and placebo lowered homocysteine with vitamins but did not measure cognition [93].

### 5.2. Vitamin D

Hiller et al. randomized 58 participants to high-dose vitamin D or placebo for 16 weeks with balance as the primary outcome; 51 completed [57]. Serum 25-hydroxyvitamin D increased, confirming exposure, but there was no between-group difference on MoCA or on a broad neuropsychological battery. Anamnart and Kitjarak randomized 60 participants with PD to vitamin D3 2000 IU/day or no additional treatment for 12 weeks and followed 60 healthy comparators [63]. Thai MMSE scores improved within both PD groups, but the between-group difference was not significant (*p* = 0.378). The absence of placebo blinding and the short duration limit inference, yet the study adds a second objective null result. The longest and largest trial was identified by the structured search: Suzuki et al. randomized 114 participants with PD to vitamin D3 1200 IU/day or placebo for 12 months, with central computerized allocation, blinding of participants, families and assessors, an authors’ intention-to-treat set of 112, and the MMSE among the article-defined primary measures [38]. Serum 25-hydroxyvitamin D rose from 22.5 to 41.7 ng/mL with supplementation. MMSE change was −0.33 ± 2.16 with vitamin D3 and +0.27 ± 1.74 with placebo (between-group *p* = 0.12), and the proportion whose MMSE did not worsen was 63.3% versus 78.2% (risk difference −0.15, 95% CI −0.32 to 0.02); the nominal direction favored placebo. The MMSE not-worsened counts and percentages imply denominators of 49 and 55, rather than the table headings of 55 and 57; the exact denominator for mean MMSE change is not explicit. The trial reported less deterioration of the Hoehn and Yahr stage with vitamin D3, modified by vitamin D receptor FokI genotype, which bears on motor progression rather than cognition. Three trials of 12 weeks to 12 months, in 58 to 114 participants, therefore show no cognitive benefit of vitamin D supplementation. Vitamin D deficiency should be treated for accepted skeletal and general-health indications; supplementation should not be presented as a PD cognitive therapy.

### 5.3. Omega-3 Fatty Acids and Vitamin E

Omega-3 fatty acids have anti-inflammatory and membrane-related plausibility. A 12-week randomized trial of flaxseed-derived omega-3 plus vitamin E in 60 participants reported improvements in UPDRS and in metabolic and inflammatory markers but measured no cognitive endpoint [94]. A 2024 systematic review of randomized omega-3 studies in PD found a small, heterogeneous literature focused on motor, mood and biomarker outcomes [95]. No controlled cognitive evidence exists in PD.

Vitamin E was tested in the DATATOP trial of 800 participants with early untreated PD, randomized in a double-blind, two-by-two factorial design to deprenyl 10 mg/day, tocopherol 2000 IU/day, both, or placebo. In the primary *NEJM* report, tocopherol did not delay disability requiring levodopa (hazard ratio 0.91, 95% CI 0.74–1.12; *p* = 0.35), with no interaction with deprenyl [36]. The companion 1994 *Neurology* brief communication reports the cognitive outcomes of the same trial [37]. Baseline cognitive assessments were available for 799 participants. Ninety participants who reached the disability endpoint or terminated before six months were excluded from annualized-change analyses; the cognitive table reports 710 participants (174 placebo, 174 tocopherol, 187 deprenyl and 175 combination). Cognitive testing was a secondary study goal, with assessments every six months over a mean of 14 ± 6 months. ANCOVA adjusted for age, gender and enrolling center, and Bonferroni correction used a threshold of *p* < 0.0033 for 15 cognitive variables. Neither treatment significantly affected cognitive change after correction. Published baseline scores and all 11 tabulated annualized-change measures are provided in Supplementary Table S3; adjusted between-group estimates and 95% CIs were not reported. This large blinded comparison did not demonstrate cognitive benefit, but the exclusion of early endpoints and terminations from cognitive analyses and the limited cognitive decline in the retained early-PD population constrain interpretation.

### 5.4. Creatine and Coenzyme Q10

The large monotherapy trials are important negative evidence. The additional cognitive, adherence, attrition and safety data are detailed in Table S2. NET-PD LS-1 randomized 1741 treated participants with early PD to creatine 10 g/day or placebo and was stopped for futility; the global outcome, which included the Symbol Digit Modalities Test, showed no benefit over a median follow-up of four years [49]. QE3 randomized 600 participants with early PD to two high doses of CoQ10 or placebo and was also stopped for futility, with no benefit on Symbol Digit performance or the broader clinical outcome [58]. Futility on a disease-progression outcome is not the same as an adequately powered test of a narrowly defined cognitive effect, and Symbol Digit performance samples processing speed and attention rather than the full PD-MCI profile. NET-PD also reported a null secondary SCOPA-Cog comparison (placebo minus creatine change −0.1, 95% CI −0.9 to 0.7) [49]. Both trials nevertheless provide strong reasons against recommending these agents for disease modification; any residual uncertainty in deficient or cognitively impaired subgroups is a research question, not a rationale for prescribing.

Li et al. randomized 75 participants with PD-MCI to creatine 5 g twice daily plus CoQ10 100 mg three times daily (*n* = 38) or matching placebo (*n* = 37) for 18 months, with MoCA as the cognitive outcome [59]. Both groups declined. The published comparisons were *p* < 0.05 at 12 months and *p* < 0.01 at 18 months, without between-group CIs; the unadjusted endpoint difference of 5.22 MoCA points at 18 months is a value calculated by the review authors from the published means (Supplementary Table S2). This difference is unusually large compared with the much larger negative monotherapy trials and requires particularly critical appraisal. Randomization was computer-generated, and blinding to allocation was described, but the blinded roles were not fully specified; participant flow, missing-data handling and the analysis population are not described, although the outcome tables retain *n* = 38 and *n* = 37; the study was single-center with no independent replication; and the two-arm design cannot separate creatine from CoQ10 effects or test synergy. Two control participants reportedly developed dementia by 18 months, an observation too sparse to support any inference about prevention. The finding is a signal of less decline on a screening instrument, not evidence of improvement or of disease modification.

### 5.5. Probiotics

Most probiotic trials in PD were designed for constipation, metabolic or neuropsychiatric outcomes. Lam et al. randomized 61 participants with PD and clinically significant anxiety to a nine-strain probiotic or placebo for 12 weeks in a double-blind trial; ten withdrew, and all 61 entered the ITT analysis [60]. The primary anxiety outcome was negative. MoCA, a secondary outcome, favored the probiotic by an adjusted mean difference of 1.1 points (95% CI 0.04–2.1; *p* = 0.043), with no parallel change in microbiome composition or systemic inflammatory markers. The lower confidence limit is close to zero, the endpoint was secondary among several, no multiplicity adjustment was reported, more impaired participants were excluded, and a one-point MoCA difference lies within the range of practice and measurement variability discussed in Section 6.2. The result is hypothesis-generating and does not establish probiotics as a treatment for PD-MCI.

Sun et al. randomized 100 participants with PD (50 per arm) to Bifidobacterium animalis subsp. lactis Probio-M8 or placebo added to conventional medication for three months [40]. After 18 intervention-period exclusions (two probiotic, 16 placebo), 82 participants contributed to clinical analyses (48 and 34); the 74 participants with complete omics samples (45 and 29) are a different analysis set. Supplementary Table S5 reports MMSE means ± SD of 25.26 ± 4.01, 26.33 ± 3.69 and 26.63 ± 3.75 with probiotic at baseline, one month and three months, versus 25.47 ± 4.37, 25.71 ± 4.32 and 25.88 ± 4.41 with placebo. The corresponding between-group *p* values were 0.71, 0.57 and 0.44; none was significant. No change-based between-group estimate or CI was reported, and differential exclusions limit inference. Zeng et al. randomized 46 participants to Bacteroides fragilis BF839 plus earthworm protein or placebo for 12 weeks [41]. In the ITT population (23 per arm), MMSE change was +0.39 ± 1.58 versus +0.57 ± 1.40 at 12 weeks (between-group *p* = 0.650); the six-week comparison was also nonsignificant (*p* = 0.335). Forty-two participants completed the study; the per-protocol results were likewise nonsignificant. Table S2 reproduces both analysis sets without confusing changes with endpoint scores. Magistrelli et al. enrolled 40 participants in a randomized trial of a three-strain Bifidobacterium formulation with fructo-oligosaccharides versus maltodextrin for 12 weeks [42]. MoCA changed from 27.47 ± 1.81 to 28.05 ± 1.68 with probiotic (within-group *p* = 0.078) and from 26.30 ± 2.77 to 25.70 ± 2.98 with placebo (*p* = 0.065); no follow-up or change-based between-group contrast was reported. Two participants dropped out, but one supplied clinical data, leaving the exact MoCA denominator unclear. In the synbiotic trial, MoCA was used for enrollment screening, but no follow-up MoCA outcome was reported; the reported cognitive outcome was the PDQ-39 cognitive-impairment domain, which does not meet this review’s eligibility criteria [35]. The trial therefore remains context, as does the probiotic–vitamin D trial [48]. Together with the borderline secondary MoCA result in the nine-strain probiotic trial [60], these studies do not establish replicated cognitive efficacy; endpoint hierarchy, multiplicity and missing-data handling remain important limitations.

### 5.6. Botanical Supplements and Combined Metabolic Activators

A double-blind phase II trial randomized 48 participants in a 2:1 ratio to combined metabolic activators (CMA; L-carnitine tartrate, N-acetylcysteine, nicotinamide riboside and L-serine) or placebo for 84 days; 43 completed [61]. MoCA, a secondary endpoint, improved from baseline in the active group, but the report states that MoCA did not differ significantly between groups at the assessed time points. Baseline scores differed numerically, and multiple subgroup and molecular analyses complicate interpretation. A significant within-group change alongside a non-significant placebo change does not constitute a treatment difference, and low baseline scores create scope for regression to the mean in either arm. This study is classified as showing no significant between-group difference at the reported time points; within-arm improvement does not establish benefit.

A triple-blind trial randomized 92 participants to saffron 100 mg/day or placebo for 12 weeks. Eighty-four completed, and all 92 were analyzed using expectation–maximization imputation. The secondary Abbreviated Mental Test (AMT) outcome favored saffron (baseline-adjusted difference +0.78 points, 95% CI 0.16–1.40; *p* = 0.01) [62]. Tables S2 and S6 provide arm scores and distinguish AMT from patient-rated PDQ-39 results. Clinical importance is uncertain: PD-MCI was not required, multiplicity was not addressed, and there is no replication. Saffron remains investigational for cognition in PD.

Two further botanical trials were identified by the structured search. Huang et al. randomly divided 80 participants with PD into compound carbidopa alone or carbidopa plus ginkgo leaf tablets for three months and reported higher MMSE and MoCA scores after treatment in the combination group (*p* < 0.05), together with lower homocysteine, malondialdehyde and neuron-specific enolase [44]. The full report specifies a random-number table and 40 participants per arm. Allocation concealment, blinding and attrition remain insufficiently reported, and numerical cognitive means and SDs are not tabulated; Figure 1 in Huang et al. [44] supplies significance markers without a numerical treatment effect or CI. MMSE and MoCA are study objectives, but their formal primary/secondary designation is not established. The favorable comparison therefore requires cautious interpretation. Shateri et al. randomized 58 participants aged 40–80 years with mild to moderate PD and a baseline MoCA of 17–30 to a Boswellia serrata extract standardized to boswellic acids (1200 mg/day) or microcrystalline-cellulose placebo for six months, with computer-generated block randomization managed by a coordinator uninvolved in assessment, sealed opaque sequentially numbered envelopes, identical capsules and blinded assessing neurologists [45]. The trial was designed around inflammatory cytokines, the only outcome in its retrospectively registered record (IRCT20170315033086N4), and UPDRS Part III; MoCA was a secondary measure. UPDRS worsened within the placebo group (15.47 ± 10.89 to 23.67 ± 11.94, *p* = 0.0008) and not within the treated group, whereas MoCA changed within neither group (22.42 ± 4.93 to 23.67 ± 3.82, *p* = 0.19; 21.00 ± 6.92 to 21.40 ± 7.33, *p* = 0.86); no between-group contrast is reported for either clinical outcome, and six-month MoCA scores were available for only 12 of 29 and 15 of 29 participants. Baseline values differ between tables 1 and 2 in Shateri et al. [45], three deaths are listed among the 14 withdrawals, one author is affiliated with the manufacturer, and the authors’ own conclusion is that cognitive stability rather than improvement was observed. Two non-randomized registry studies of Pycnogenol (French maritime pine bark extract) reported lower physician-rated 0–4 scores for cognitive symptoms with supplementation than with standard management alone over four and eight weeks [46,47]; without random allocation or an objective instrument they are context, not evidence. A fermented papaya preparation, a functional food marketed for its antioxidant properties, was tested in a randomized, double-blind, placebo-controlled crossover trial: 39 participants with Hoehn and Yahr stage 1–2 PD received 9 g/day of the preparation or placebo for 180 days and then the alternate treatment for 180 days, with MoCA, Frontal Assessment Battery, UPDRS, fatigue and PDQ-8 scores recorded at each visit [43]. The analysis pooled the two active periods and the two placebo periods and compared scores before and after each: MoCA rose during active treatment (paired *p* = 0.015) but not during placebo (*p* = 0.73), the Frontal Assessment Battery changed in neither, and no between-treatment contrast, score means, carry-over analysis, washout, registration or attrition is reported; the manufacturer and distributor provided financial support. The result is a within-period change on one of two cognitive instruments among nine scales, not evidence of a treatment effect. The botanical and functional-food literature therefore consists of one small multiple-outcome screening difference (saffron), one weakly reported positive trial (ginkgo), two within-group analyses without a between-treatment contrast (boswellic acids, fermented papaya) and two registry studies; none establishes cognitive efficacy.

In summary, supplement evidence for cognition in PD comprises three null vitamin D trials, one null niacin trial and one randomized niacin phase with descriptive cognitive data but no comparative test, the large negative DATATOP tocopherol comparison, two large negative monotherapy trials, one unreplicated 18-month combination trial with incompletely reported participant flow, one borderline secondary probiotic result and three probiotic trials without a between-group cognitive effect, one metabolic activator trial with no significant between-group difference at the reported time points, one small multiple-outcome saffron difference, one weakly reported positive ginkgo trial and two within-group-only analyses of boswellic acids and fermented papaya. No supplement has replicated objective cognitive benefit, and no supplement trial has tested progression to PDD as an endpoint.

## 6. Evidence Synthesis and Clinical Implications

**Figure 2:**
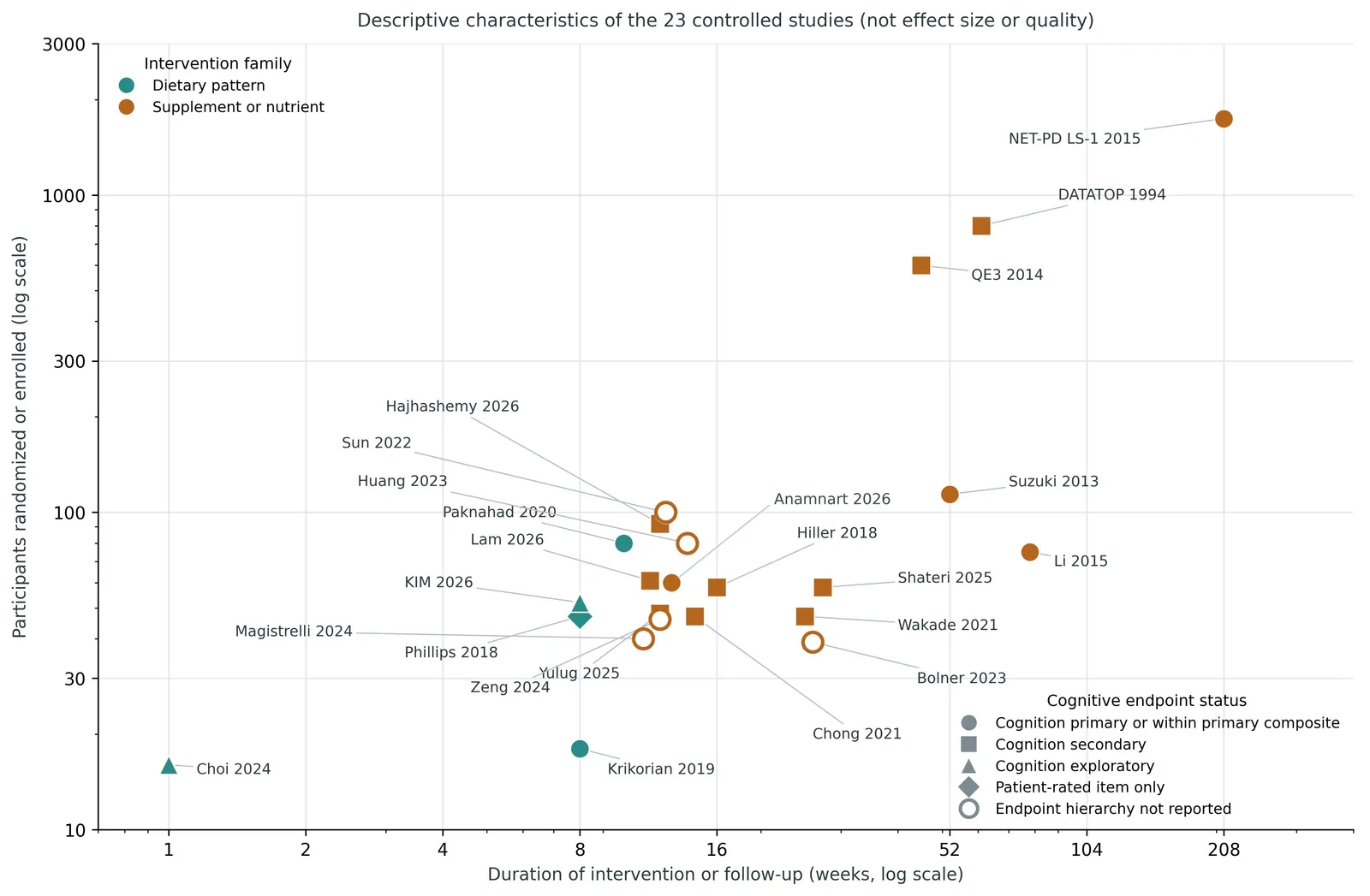
Descriptive characteristics of the 23 controlled studies [36,37,38,39,40,41,42,43,44,45,49,50,51,53,54,55,56,57,58,59,60,61,62,63]. Marker color indicates the intervention family (dietary pattern or supplement), and marker shape indicates the cognitive endpoint status as reported in Table 3 (filled circle, primary or within the primary composite; filled square, secondary; filled triangle, exploratory; filled diamond, patient-rated item only; open circle, endpoint hierarchy not reported). Both axes are logarithmic. Positions describe size and duration, not effect size or quality. Sun: 100 randomized, 82 analyzed clinically. Krikorian: 18 enrolled, 14 analyzed. Choi: one-week comparison. QE3 and NET-PD LS-1: mean and median follow-up, respectively. KIM and Bolner: one crossover period. Chong: 47 randomized (16/16/15), 46 completed three months; its uncontrolled follow-up is not plotted.

The 23 controlled studies form a hierarchy rather than a tally. At the top are three large, blinded trials in early PD that were negative for creatine, CoQ10 and tocopherol on outcomes that included cognitive testing [37,49,58], and a 12-month vitamin D trial with central randomization and an article-defined primary MMSE outcome that was also null [38]. The DATATOP cognitive analysis excluded 90 early endpoints or terminations and included 710 participants, rather than all 800 randomized [37]. Below them are two small trials that favored the intervention on an objective test but used completer analyses (Krikorian: 14 of 18; Paknahad: 70 of 80) [53,54], and one 18-month combination trial with incompletely reported flow [59]. Alongside these are secondary or multiple-outcome between-group differences on screening instruments whose lower confidence limits approach zero (probiotic: MoCA +1.1 points, 95% CI 0.04–2.1; saffron: AMT +0.78 points, 95% CI 0.16–1.40) [60,62], one weakly reported positive ginkgo trial [44], and further reports without demonstrated comparative cognitive benefit [39,40,41,42,43,45,50,56,57,63]. These include nonsignificant between-group comparisons, within-group-only analyses and a rated-symptom study; they are not equivalent negative cognitive trials. Chong’s three-month randomized phase adds arm-level cognitive data without a comparative test [51]. Counting six studies with a favorable result and seventeen without would misrepresent this evidence because these categories carry different weight. No included trial established durable functional benefit or reduced conversion to PDD. Figure 2 summarizes the descriptive characteristics of the 23 studies.

The three therapeutic claims yield different conclusions. Nutritional rescue is supported as clinical care by deficiency and malnutrition guidance, but the included trials do not establish that correcting a documented deficit prevents PDD [33,82]. Short-term cognitive performance is the claim most directly addressed by the Mediterranean and PD-MCI ketogenic pilots, and the probiotic and saffron findings extend this hypothesis through secondary or multiple-outcome screening measures [44,53,54,60,62]. Progression is addressed more closely by the 18-month combination study and the longer monotherapy and vitamin trials, with one unreplicated positive combination result and four negative results from trials of four months or longer [37,38,49,58,59]. Neither a short-term screening gain nor successful biochemical correction should be described as disease modification.

### 6.1. Biomarkers, Target Engagement and Cognitive Efficacy

**Figure 3:**
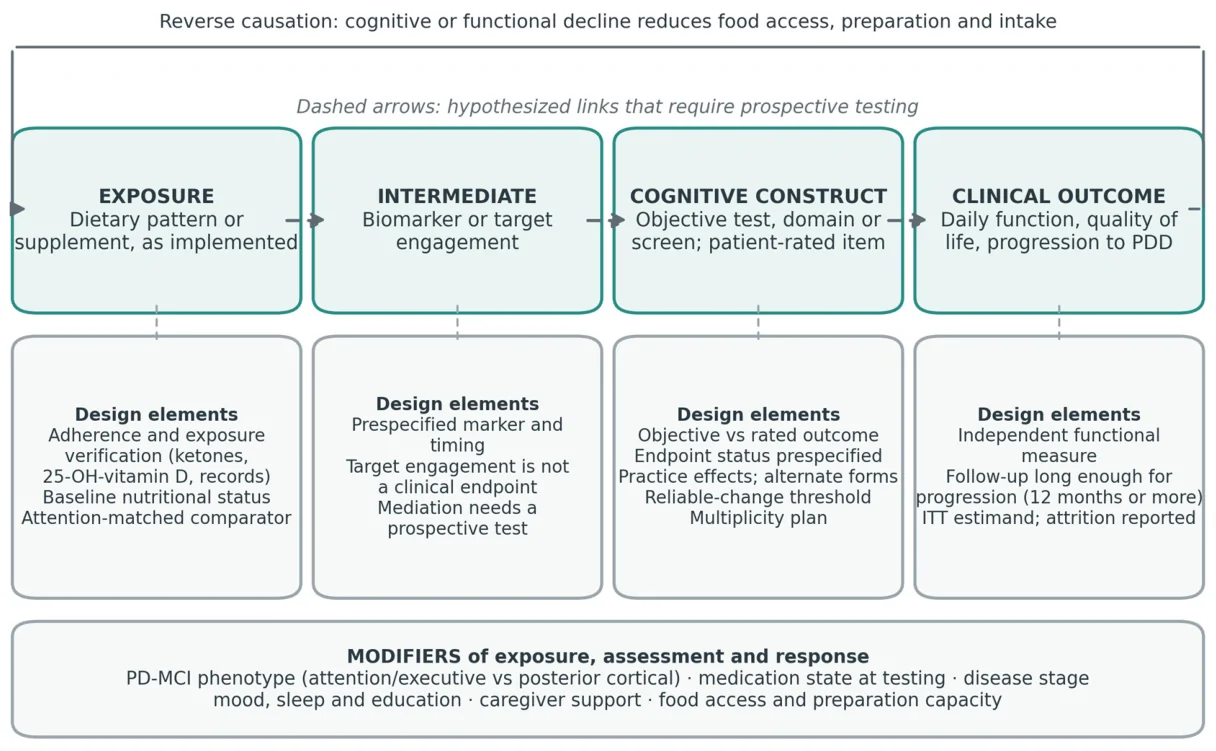
A framework for testing nutritional effects on cognition in Parkinson’s disease. Dashed arrows represent hypothesized links that require prospective testing. The solid arrow indicates potential reverse causation. Design elements listed under each stage determine whether a study can test the corresponding link. The diagram is conceptual and does not establish mediation. PD-MCI, Parkinson’s disease–mild cognitive impairment; PDD, Parkinson’s disease dementia; ITT, intention-to-treat.

Nutrition could influence cognition through cardiometabolic and vascular health, nutritional reserve, one-carbon metabolism and gut–immune or mitochondrial signaling (Section 3). The existence of these pathways does not show that an intervention reaches the relevant target at a sufficient dose, or that a biomarker change translates into cognitive benefit. Three statements follow. First, target engagement, such as a rise in serum 25-hydroxyvitamin D, a fall in homocysteine, documented ketosis or a shift in microbiome composition, is not a clinical endpoint. Second, a short-term difference on a screening score is not a functional benefit. Third, neither demonstrates slower progression to PDD. Biomarkers should be treated as mediators only when the intervention produces a prespecified change that precedes the cognitive outcome and the biomarker–cognition relationship is analyzed without selective reporting. Figure 3 sets out the causal chain, from dietary exposure through hypothesized intermediates to the cognitive construct measured and the clinical outcome of interest, together with the design elements that determine whether a study can test each link.

### 6.2. Measuring Cognition: Screening Scores, Practice Effects and Meaningful Change

Global screens such as the MoCA, MMSE and AMT are practical for detecting broad change, and the MoCA is recommended as a minimum cognitive screen in PD trials where cognition is not the primary outcome [96,97]. They are not interchangeable with an MDS Level II PD-MCI assessment. In pooled data from 467 people with PD, the C-statistic for dementia prediction was 0.81 for Level II assessment compared with 0.72 for MMSE and 0.70 for MoCA [98], and MoCA subsection scores are not validated as stand-alone measures of specific domains in PD [99]. A single patient-rated item from MDS-UPDRS Part I or a quality-of-life scale is further removed still from objective performance. Composite executive endpoints such as the Parkinson’s Disease Composite of Executive Functioning are one attempt to build a more sensitive trial measure but still require validation across languages and phenotypes [100].

Repeated administration of the same instrument over 8–16 weeks produces practice effects. Meta-analyses of neuropsychological assessment show gains on retest that vary by test, interval and age [101], and practice-effect-corrected reliable-change indices have been developed for PD specifically because serial testing otherwise overstates improvement [102]. Randomization distributes practice effects across arms, but it does not remove them from within-group change, and it does not protect a completer analysis in which the arms differ in who returns for retesting. Among the included studies, the KIM, nine-strain probiotic, niacin, 12-month vitamin D and saffron trials reported ITT analyses explicitly [38,39,50,60,62]; the other studies used the same screening instrument at baseline and follow-up and did not report using alternate forms. No minimal clinically important difference for the MoCA or the AMT has been established in PD-MCI within the sources identified for this review, so the 1.1-point probiotic and 0.78-point saffron differences cannot be judged clinically meaningful; their size alone does not establish clinical importance, and group-mean differences should not be equated with the integer scoring resolution of an individual item. A confirmatory trial should pair a prespecified cognitive effect with an independently assessed functional outcome and a responder definition justified before unblinding.

### 6.3. Small Studies, Attention, Adherence and Attrition

Small samples yield both exaggerated positive estimates and inconclusive negative findings. The evidence set is too heterogeneous and selectively assembled for a formal small-study-effect analysis; the concentration of favorable findings in the smallest studies is a reason for caution, not proof of publication bias. For any trial, the CI and a prespecified meaningful-change threshold matter more than whether the *p* value falls just above or below 0.05, and “no significant difference” should not be rewritten as “no effect”.

Dietary trials test a package of food changes, counseling, attention and practical support, so greater contact in the intervention arm can improve engagement, medication routines or meal regularity and contribute to apparent benefit. An attention-matched comparator addresses part of this problem; blinded outcome assessors, documented medication state, standardized testing conditions and alternate test forms reduce further bias, although expectancy effects remain when the diet itself cannot be masked. Adherence and attrition are outcomes as well as threats to validity. An ITT analysis estimates the consequence of offering the program as tested; a completer analysis describes those able to sustain it. Excluding non-adherent participants after randomization, as in the Mediterranean trial, converts a randomized comparison into an observational one and can exaggerate efficacy [53]. Post-randomization weight change may be part of the treatment pathway, so adjusting for it automatically can remove a true effect or introduce bias; the intended estimand should be specified in advance.

### 6.4. Global Burden, Dietary Patterns and the Limits of Extrapolation

The burden of PD is rising fastest in regions undergoing dietary transition. East Asia and South Asia are projected to carry the largest numbers of cases in 2050, and western sub-Saharan Africa the largest relative increase [1,2]. Prospective cohorts in North America and Europe associate prudent, Mediterranean-type or MIND-type patterns with lower PD risk and fewer prodromal features, and Western patterns with higher risk [13,16,17,19]. These associations are relevant to prevention research and to public-health messaging, but they cannot be transferred to cognition in established PD. Risk cohorts measure diet decades before diagnosis in people without disease; the cognitive trials reviewed here enrolled people with established PD for weeks to months. Dietary-pattern data from the Middle East, Africa and most of Asia are sparse. The small Iranian Mediterranean-diet trial [53] provides preliminary cognitive evidence, but the effectiveness and generalizability of culturally adapted dietary patterns across these regions remain uncertain. The appropriate PD-specific question remains whether a defined dietary intervention provides cognitive benefit over a credible, adequately supported comparator in the population that will use it.

### 6.5. From Evidence to Clinical Action

The clinical task is not to prescribe an unproven “brain diet”; it is to identify nutritional problems that can worsen resilience, medication response or test performance. Assessment should include unintended weight loss and weight trajectory, dietary intake, swallowing, constipation and hydration, ability to shop and prepare food, caregiver support and reproducible levodopa–meal interactions. Laboratory testing should follow clinical indications rather than a universal panel. Neurology nutrition guidance supports attention to nutritional risk, dysphagia, protein–levodopa interactions and adequate energy and protein across the disease course [33,82].

**Figure 4:**
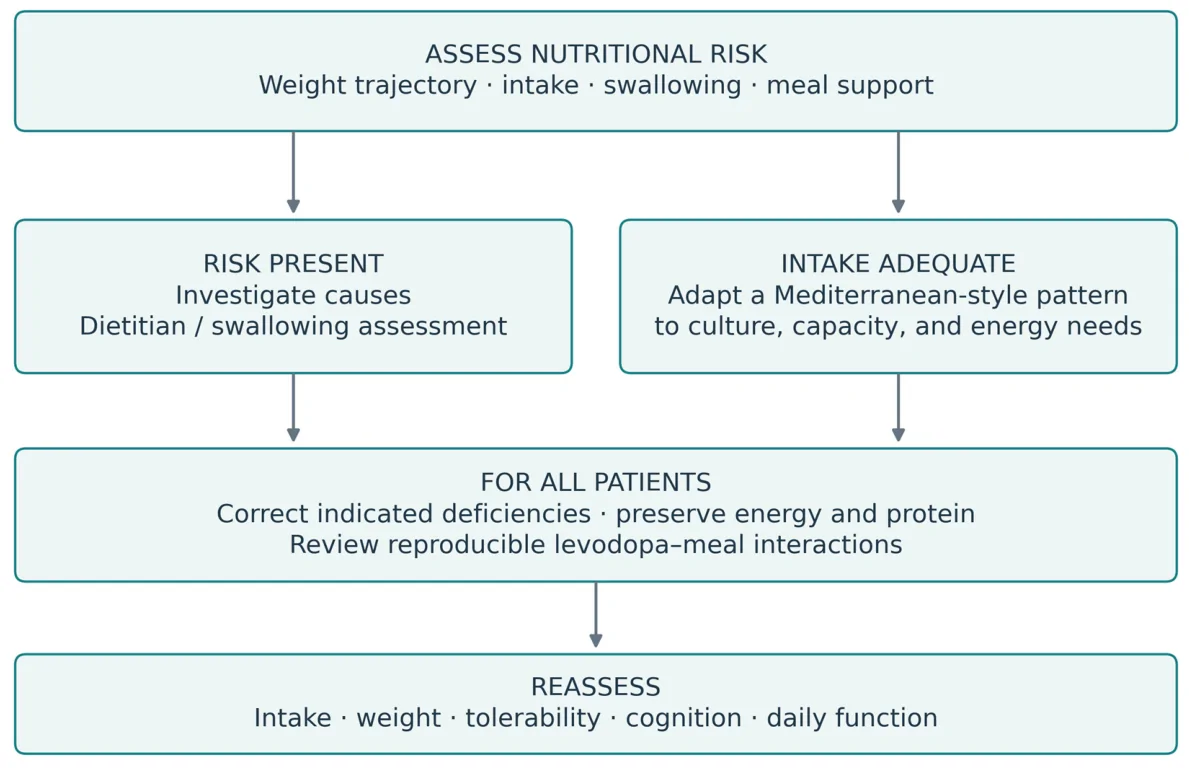
Supportive nutritional care when cognition is a concern in Parkinson’s disease. The approach synthesizes guidance [33,82]; it is not a validated algorithm or a treatment for cognitive decline.

For a patient without urgent nutritional risk, a culturally adapted Mediterranean-style pattern is a reasonable default because it supports dietary quality and cardiovascular health [75], not because PD-specific cognitive protection has been established. Deficiencies should be corrected for accepted indications. Protein redistribution may be considered when meal-related levodopa failure is reproducible, preferably with dietetic supervision and preservation of total protein [90,91]. Restrictive ketogenic regimens, high-dose supplements, CMA and botanical supplements should remain research interventions for cognition. Weight, intake, adverse effects, cognition and function should be reassessed rather than assuming that biochemical target engagement equals patient benefit (Figure 4).

Implementation should reflect the patient’s capacity to obtain, prepare and consume the recommended foods. For a person with executive impairment, a simpler meal plan, a recurring shopping list, prepared portions or caregiver assistance may be more useful than detailed nutrient targets. In dysphagia, texture and swallowing safety take priority; in patients losing weight, energy enrichment takes priority over restriction [33]. Counseling should specify what will be monitored and what would prompt revision: continued weight loss, difficulty swallowing, reduced intake, worsening constipation or an impractical medication–meal schedule. Cognitive scores should be interpreted alongside daily function and caregiver observations rather than used alone to justify continuing an expensive supplement.

Table 4 summarizes evidence-calibrated counseling statements. Each row cites its sources, and the footnote distinguishes recommendations that rest on general PD nutrition guidance from those that rest on cognition-specific trial evidence. The statements are deliberately conservative and prioritize avoidance of harm in patients vulnerable to weight loss, dysphagia, constipation, polypharmacy and financial burden.

In summary, the synthesis supports active, individualized nutritional care and a Mediterranean-style default, while withholding any claim that a diet or supplement treats cognitive impairment in PD. The evidence hierarchy, the measurement limits of screening instruments and the global context all point the same way: toward confirmatory trials rather than clinical adoption.

## 7. Priorities for Future Trials

A confirmatory efficacy trial should enroll participants with PD-MCI defined by MDS criteria, stratify by cognitive phenotype and nutritional status, and justify a sample size for one prespecified cognitive primary endpoint. Global screens may support eligibility or secondary analysis, but domain-level cognition and function should anchor inference, with alternate test forms and practice-effect-corrected change thresholds specified in advance [102]. Follow-up should be long enough to separate practice effects and transient symptomatic change from delayed decline, with extension phases for durability and PDD progression where feasible.

Interventions should be manualized but culturally adaptable, with an attention-matched comparator, objective adherence or exposure measures (for example, blood β-hydroxybutyrate for ketogenic diets, serum 25-hydroxyvitamin D for vitamin D) and prespecified handling of weight change. Core safety outcomes should include unintentional weight loss, lean mass, falls, constipation, dysphagia, cardiometabolic measures and medication interactions. Recruitment should represent women and men, varied educational and cultural backgrounds, frailty states and common comorbidities, and should extend to the regions where PD burden is growing fastest. Protocols and statistical analysis plans should define the primary comparison, the cognitive multiplicity strategy, missing-data assumptions and sensitivity analyses before unblinding, and biomarker analyses should test temporal and mechanistic hypotheses without treating target engagement as a substitute for clinical benefit.

A pragmatic next trial could evaluate a dietitian-supported Mediterranean-style program in well-characterized PD-MCI against equally intensive general healthy-eating support. Follow-up of 12–18 months would allow assessment beyond immediate practice effects, with a longer extension for progression. One validated cognitive composite should be primary, with function, nutritional status, adherence and adverse events as prespecified secondary outcomes, and the sample size should rest on a clinically justified between-group difference and realistic attrition rather than on an inflated pilot effect. Participants with deficiencies should receive indicated correction in both arms. Different questions require different estimands: a nutritional rescue study asks whether correcting inadequate intake improves cognition and function; an efficacy study asks whether the assigned intervention changes cognitive decline; an implementation study asks whether the program is sustainable in usual care. Caregiver time, food affordability and cost should be measured alongside biological adherence, because a modest cognitive effect has little value if the intervention imposes substantial burden or accelerates nutritional risk.

## 8. Limitations of This Review

This review has several limitations. Retrieval for the original submission was targeted rather than protocol-driven, and intervention selection and interpretation depended substantially on the authors’ judgment because no protocol was preregistered; citation-selection bias is therefore possible, and the structured search added for this revision reduces but does not eliminate it. One author screened and extracted the data, and Amaal Aldakheel verified the evidence tables against the available sources; there was no independent duplicate screening. Eligibility was restricted to English-language reports. This restriction most likely excludes trials from regions where PD burden is rising fastest; at least one Chinese-language ketogenic RCT with motor outcomes is known to exist [24], and if non-English reports differ systematically in direction or quality the restriction could bias the evidence set in either direction. No unpublished data were requested from investigators. All included studies were assessed from full reports. FThree nonincluded reports were assessed from abstract-level material because their full reports had not been retrieved, so their full-report eligibility cannot be certified. NR in the evidence tables denotes information absent from the inspected report. Trial-registration identifiers do not independently establish prospective endpoint prespecification. No formal overall risk-of-bias rating, pooled estimate or assessment of publication bias was undertaken. Heterogeneity in PD stage, baseline nutrition, cognitive phenotype, intervention, comparator and outcome limits cross-trial comparison, and the exclusion of more impaired participants in several trials limits applicability to PDD. Participant overlap between the two niacin protocols is not explicitly resolved; study-level enrollment sums should not be interpreted as verified counts of unique individuals. The descriptive categories in Table 3 and Figure 2 are not treatment rankings. The clinical statements in Table 4 synthesize cited evidence and guidance; they are not a formally developed practice guideline.

## 9. Conclusions

No dietary pattern or nutritional supplement has shown independently replicated, durable cognitive efficacy in PD, and none has been shown to prevent PDD. Small trials support testable hypotheses about short-term performance; the three largest trials did not demonstrate benefit on their reported cognitive outcomes. Nutritional adequacy, correction of documented deficiency, dysphagia care and individualized levodopa–meal timing remain the clinical priorities, and a culturally adapted Mediterranean-style pattern can support these goals. Confirmatory trials should separate rescue, performance and progression, verify exposure, and demonstrate cognitive effects that are precise, functionally meaningful, sustainable and safe.

## Figures and Tables

**Table 4:** Evidence-calibrated counseling for nutrition and cognition in Parkinson’s disease.

Strategy	What the Evidence Supports	What Can Be Said in Clinic	Safeguards	Basis ^†^
Mediterranean-style pattern	One short cognitive RCT with a positive completer analysis [53]; cardiovascular benefit in the general population [75]; PD-risk associations are indirect [19]	Reasonable default for dietary quality and cardiovascular health; cognitive protection in PD is unproven	Adapt to culture, diabetes, dysphagia, food access and energy needs; monitor weight	G + C
Weight and nutritional adequacy	Malnutrition and dysphagia are common [30,31]; early weight loss predicts faster cognitive decline, causality uncertain [32]	Prevent and investigate unintentional weight loss; screen intake, swallowing, constipation and meal support	Dietitian referral; serial weight; protein and energy enrichment; reversible medical causes [33,82]	G
Vitamin B12, folate and niacin	Supplementation corrects deficiency and lowers homocysteine [77,93]; one six-month niacin trial showed no cognitive difference [39]; prevention of cognitive decline has not been demonstrated	Test and replace when deficient or clinically indicated; do not promise cognitive benefit	Interpret B12 with methylmalonic acid when needed; consider renal function and levodopa exposure	G
Vitamin D	Three PD trials of 12 weeks to 12 months showed no between-group cognitive benefit [38,57,63]	Treat deficiency for accepted skeletal and general indications, not as PD cognitive therapy	Avoid unsupervised high doses; monitor calcium and renal risk when appropriate	G + C
Protein redistribution	May improve levodopa-related motor fluctuations [90,91]; no cognitive evidence	Use only for reproducible dose–meal interactions while preserving total daily protein	Dietitian supervision; avoid chronic low-protein intake, weight loss and sarcopenia	G
Ketogenic diets	One very small positive PD-MCI pilot [54]; later objective findings null and adherence difficult [50,56]	Experimental for cognition; not routine care	Research or specialist supervision; monitor weight, lipids, constipation, renal risk, micronutrients and medication effects	C
Creatine, CoQ10, vitamin E, omega-3, probiotics	Large creatine, CoQ10 and tocopherol trials did not demonstrate cognitive benefit [37,49,58]; probiotic results comprise a borderline secondary difference, nonsignificant MMSE comparisons and a within-group-only report [40,41,42,60]; no cognitive omega-3 evidence [95]	Do not recommend to prevent PD cognitive decline	Review interactions, dose, product quality, cost, renal and hepatic status and bleeding risk	C
CMA and botanical supplements (saffron, ginkgo, boswellic acids, fermented papaya)	Saffron: small screening difference among many outcomes [62]; ginkgo: positive but weakly reported trial [44]; boswellic acids and fermented papaya: within-period changes without a between-treatment MoCA contrast [43,45]; CMA: within-group improvement without between-group MoCA difference [61]	Investigational for cognition; not disease-modifying treatment	Use only within research or after clinician review; check interactions, product quality, metabolic or renal risk and cost	C

^†^ Basis: G, recommendation rests on general PD nutrition guidance (nutritional adequacy, dysphagia care, deficiency correction) [33,82]; C, statement rests on the cognition-specific controlled evidence reviewed here. Rows marked G + C combine both. None of the cognition-specific evidence establishes efficacy. Abbreviations: CMA, combined metabolic activators; CoQ10, coenzyme Q10; MoCA, Montreal Cognitive Assessment; PD, Parkinson’s disease; PD-MCI, Parkinson’s disease mild cognitive impairment; RCT, randomized controlled trial; MMSE, Mini-Mental State Examination.

## Data Availability

The data supporting this review are available in the cited publications and their Supplementary Materials. The study-level extracted data and appraisal information are provided in this article and its Supplementary Materials.

## Supplementary Materials

The following supporting information can be downloaded at https://www.mdpi.com/article/10.3390/nu18203436/s1, Supplementary S1: Search strategy, databases, dates and record counts; Table S1: Domain-level appraisal of the 23 controlled studies; Table S2: Cognitive Score Sequences, Adherence, Attrition and Adverse Events; Table S3: DATATOP Cognitive Scores and Annualized Changes; Table S4: Hiller 2018 cognitive change scores; Table S5: Choi 2024 Cognitive Scores During the Randomized Inpatient Phase; Table S6: Hajhashemy 2026 Cognitive Outcomes.
