# Supplementary material for: Dietary Patterns and Nutritional Interventions for Cognitive Impairment in Parkinson’s Disease: A Critical Narrative Review
Source: Nutrients. 2026 Oct 20;18(20):3436. doi: 10.3390/nu18203436 (PMC13708064; doi:10.3390/nu18203436)
Supplement: Supplementary file 1 [file nutrients-18-03436-s001.zip › nutrients-4581545-supplementary.pdf]

# Supplementary Materials

*Dietary Patterns and Nutritional Interventions for Cognitive Impairment in Parkinson's Disease: A Critical Narrative Review*

Amaal Aldakheel and Najeeb Qadi

## Supplementary S1. Search strategy, databases, dates and record counts

Dietary Patterns and Nutritional Interventions for Cognitive Impairment in Parkinson's Disease: A Critical Narrative Review. Searches were run on 29 September 2026 through the King Faisal Specialist Hospital and Research Centre library (PubMed through its public interface). Four concept blocks were combined in PubMed, Embase and Web of Science: (A) Parkinson's disease; (B) cognition or cognitive outcome; (C) dietary pattern, food-based or nutritional-supplement intervention; (D) controlled intervention design. CENTRAL, which indexes controlled trials only, was searched with blocks A to C as free-text lines. Limits: human studies, English language, no date limit (database inception to the search date). Embase additionally excluded conference abstracts. Retrieval for the original submission (targeted retrieval with reference-list checking; publication cutoff 31 August 2026) is described in Section 2.2 of the main text. Trial registries and grey literature were not searched.

Databases, interfaces, dates and record counts.

| Database | Interface, edition or issue | Date run | Records retrieved |
|---|---|---|---|
| PubMed | pubmed.ncbi.nlm.nih.gov (MEDLINE and PubMed-only records) | 29 September 2026 | 320 |
| Embase | embase.com | 29 September 2026 | 927 |
| Web of Science Core Collection | All editions | 29 September 2026 | 1,583 |
| Cochrane CENTRAL | Cochrane Library, Issue 8 of 12, August 2026 | 29 September 2026 | 373 |
| Total | | | 3,203 |
| After removal of duplicates | | | 2,575 |

Duplicates were removed by matching DOI, then PubMed identifier, then normalized title (628 removed). Recorded counts are 2,575 screened, 2,542 excluded at title/abstract and 33 selected reports. Twenty-nine were assessed from full reports (21 included and eight not included). Four other reports were not included on the basis of abstract-level information because their full reports remained unavailable; they are not represented as verified full-text exclusions. The 21 database-derived inclusions plus two full reports identified through targeted retrieval give 23 included studies, all assessed from full reports. Figure 1 shows these routes.

### *PubMed*

```
("Parkinson Disease"[Mesh] OR parkinson*[tiab]) AND ("Cognition"[Mesh] OR "Neurocognitive Disorders"[Mesh] OR
"Cognitive Dysfunction"[Mesh] OR "Neuropsychological Tests"[Mesh] OR "Mental Status and Dementia Tests"[Mesh]
OR "Memory"[Mesh] OR "Executive Function"[Mesh] OR cognit*[tiab] OR dementia[tiab] OR "mild cognitive
impairment"[tiab] OR MCI[tiab] OR neuropsycholog*[tiab] OR memory[tiab] OR "executive function"[tiab] OR
"executive functions"[tiab] OR attention[tiab] OR MoCA[tiab] OR "Montreal Cognitive Assessment"[tiab] OR
MMSE[tiab] OR "mini-mental"[tiab] OR "mini mental"[tiab]) AND ("Diet"[Mesh] OR "Diet Therapy"[Mesh] OR
"Nutrition Therapy"[Mesh] OR "Dietary Supplements"[Mesh] OR "Micronutrients"[Mesh] OR "Vitamins"[Mesh] OR
"Vitamin D"[Mesh] OR "Vitamin B 12"[Mesh] OR "Folic Acid"[Mesh] OR "Homocysteine"[Mesh] OR "Creatine"[Mesh]
OR "Ubiquinone"[Mesh] OR "Probiotics"[Mesh] OR "Prebiotics"[Mesh] OR "Synbiotics"[Mesh] OR "Fatty Acids,
Omega-3"[Mesh] OR "Fish Oils"[Mesh] OR "Crocus"[Mesh] OR "Curcumin"[Mesh] OR "Polyphenols"[Mesh] OR
"Fasting"[Mesh] OR "Caloric Restriction"[Mesh] OR "Ketone Bodies"[Mesh] OR "Dietary Proteins"[Mesh] OR
diet[tiab] OR diets[tiab] OR dietary[tiab] OR nutrition*[tiab] OR nutrient*[tiab] OR nutraceutical*[tiab] OR
supplement*[tiab] OR Mediterranean[tiab] OR "MIND diet"[tiab] OR ketogenic[tiab] OR ketone*[tiab] OR
ketosis[tiab] OR "medium chain triglyceride"[tiab] OR "medium chain triglycerides"[tiab] OR "medium-chain
triglyceride"[tiab] OR "medium-chain triglycerides"[tiab] OR "low protein"[tiab] OR "low-protein"[tiab] OR
"protein redistribution"[tiab] OR "protein restriction"[tiab] OR fasting[tiab] OR "caloric restriction"[tiab]
OR "calorie restriction"[tiab] OR "time restricted"[tiab] OR "time-restricted"[tiab] OR vitamin*[tiab] OR
cholecalciferol[tiab] OR cobalamin[tiab] OR cyanocobalamin[tiab] OR methylcobalamin[tiab] OR folate[tiab] OR
"folic acid"[tiab] OR homocysteine[tiab] OR creatine[tiab] OR "coenzyme Q10"[tiab] OR "coenzyme Q"[tiab] OR
ubiquinone[tiab] OR ubiquinol[tiab] OR CoQ10[tiab] OR "nicotinamide riboside"[tiab] OR probiotic*[tiab] OR
```

```
prebiotic*[tiab] OR synbiotic*[tiab] OR "omega-3"[tiab] OR "omega 3"[tiab] OR docosahexaenoic[tiab] OR
DHA[tiab] OR "fish oil"[tiab] OR saffron[tiab] OR crocin[tiab] OR crocetin[tiab] OR curcumin[tiab] OR
polyphenol*[tiab] OR flavonoid*[tiab] OR "green tea"[tiab] OR epigallocatechin[tiab] OR EGCG[tiab] OR
"metabolic activator"[tiab] OR "metabolic activators"[tiab] OR "nutritional ketosis"[tiab] OR "ketone
ester"[tiab] OR "ketone esters"[tiab]) AND ("Randomized Controlled Trial"[pt] OR "Controlled Clinical
Trial"[pt] OR "Clinical Trial"[pt] OR randomized[tiab] OR randomised[tiab] OR randomly[tiab] OR placebo[tiab]
OR "controlled trial"[tiab] OR "controlled study"[tiab] OR controlled[tiab] OR "control group"[tiab] OR
"pilot trial"[tiab] OR "pilot study"[tiab] OR crossover[tiab] OR "cross-over"[tiab] OR "cross over"[tiab])
NOT (animals[mh] NOT humans[mh]) AND english[la]
```

### *Embase (embase.com)*

```
('parkinson disease'/exp OR parkinson*:ti,ab,kw) AND ('cognition'/exp OR 'cognitive defect'/exp OR
'dementia'/exp OR 'neuropsychological test'/exp OR 'memory'/exp OR 'executive function'/exp OR
cognit*:ti,ab,kw OR dementia:ti,ab,kw OR 'mild cognitive impairment':ti,ab,kw OR mci:ti,ab,kw OR
neuropsycholog*:ti,ab,kw OR memory:ti,ab,kw OR 'executive function':ti,ab,kw OR 'executive
functions':ti,ab,kw OR attention:ti,ab,kw OR moca:ti,ab,kw OR 'montreal cognitive assessment':ti,ab,kw OR
mmse:ti,ab,kw OR 'mini-mental':ti,ab,kw OR 'mini mental':ti,ab,kw) AND ('diet'/exp OR 'diet therapy'/exp OR
'diet supplementation'/exp OR 'dietary supplement'/exp OR 'micronutrient'/exp OR 'vitamin'/exp OR 'folic
acid'/exp OR 'homocysteine'/exp OR 'creatine'/exp OR 'ubidecarenone'/exp OR 'probiotic agent'/exp OR
'prebiotic agent'/exp OR 'omega 3 fatty acid'/exp OR 'fish oil'/exp OR 'curcumin'/exp OR 'polyphenol'/exp OR
'fasting'/exp OR 'caloric restriction'/exp OR 'ketone body'/exp OR 'ketogenic diet'/exp OR 'mediterranean
diet'/exp OR 'low protein diet'/exp OR 'nutraceutical'/exp OR diet:ti,ab,kw OR diets:ti,ab,kw OR
dietary:ti,ab,kw OR nutrition*:ti,ab,kw OR nutrient*:ti,ab,kw OR nutraceutical*:ti,ab,kw OR
supplement*:ti,ab,kw OR mediterranean:ti,ab,kw OR 'mind diet':ti,ab,kw OR ketogenic:ti,ab,kw OR
ketone*:ti,ab,kw OR ketosis:ti,ab,kw OR 'medium chain triglyceride':ti,ab,kw OR 'medium chain
triglycerides':ti,ab,kw OR 'medium-chain triglyceride':ti,ab,kw OR 'medium-chain triglycerides':ti,ab,kw OR
'low protein':ti,ab,kw OR 'low-protein':ti,ab,kw OR 'protein redistribution':ti,ab,kw OR 'protein
restriction':ti,ab,kw OR fasting:ti,ab,kw OR 'caloric restriction':ti,ab,kw OR 'calorie restriction':ti,ab,kw
OR 'time restricted':ti,ab,kw OR 'time-restricted':ti,ab,kw OR vitamin*:ti,ab,kw OR cholecalciferol:ti,ab,kw
OR cobalamin:ti,ab,kw OR cyanocobalamin:ti,ab,kw OR methylcobalamin:ti,ab,kw OR folate:ti,ab,kw OR 'folic
acid':ti,ab,kw OR homocysteine:ti,ab,kw OR creatine:ti,ab,kw OR 'coenzyme q10':ti,ab,kw OR 'coenzyme
q':ti,ab,kw OR ubiquinone:ti,ab,kw OR ubiquinol:ti,ab,kw OR coq10:ti,ab,kw OR 'nicotinamide
riboside':ti,ab,kw OR probiotic*:ti,ab,kw OR prebiotic*:ti,ab,kw OR synbiotic*:ti,ab,kw OR 'omega-3':ti,ab,kw
OR 'omega 3':ti,ab,kw OR docosahexaenoic:ti,ab,kw OR dha:ti,ab,kw OR 'fish oil':ti,ab,kw OR saffron:ti,ab,kw
OR crocin:ti,ab,kw OR crocetin:ti,ab,kw OR curcumin:ti,ab,kw OR polyphenol*:ti,ab,kw OR flavonoid*:ti,ab,kw
OR 'green tea':ti,ab,kw OR epigallocatechin:ti,ab,kw OR egcg:ti,ab,kw OR 'metabolic activator':ti,ab,kw OR
'metabolic activators':ti,ab,kw OR 'nutritional ketosis':ti,ab,kw OR 'ketone ester':ti,ab,kw OR 'ketone
esters':ti,ab,kw) AND ('randomized controlled trial'/exp OR 'controlled clinical trial'/exp OR 'crossover
procedure'/exp OR 'double blind procedure'/exp OR 'single blind procedure'/exp OR 'placebo'/exp OR 'pilot
study'/exp OR random*:ti,ab,kw OR placebo*:ti,ab,kw OR 'controlled trial':ti,ab,kw OR 'controlled
study':ti,ab,kw OR 'control group':ti,ab,kw OR 'pilot trial':ti,ab,kw OR 'pilot study':ti,ab,kw OR
crossover:ti,ab,kw OR 'cross over':ti,ab,kw) AND [humans]/lim AND [english]/lim NOT [conference abstract]/lim
```

### *Web of Science Core Collection*

```
TS=(parkinson*) AND TS=(cognit* OR dementia OR "mild cognitive impairment" OR MCI OR neuropsycholog* OR
memory OR "executive function" OR "executive functions" OR attention OR MoCA OR "Montreal Cognitive
Assessment" OR MMSE OR "mini-mental" OR "mini mental") AND TS=(diet OR diets OR dietary OR nutrition* OR
nutrient* OR nutraceutical* OR supplement* OR Mediterranean OR "MIND diet" OR ketogenic OR ketone* OR ketosis
OR "medium chain triglyceride" OR "medium chain triglycerides" OR "medium-chain triglyceride" OR "medium-
chain triglycerides" OR "low protein" OR "low-protein" OR "protein redistribution" OR "protein restriction"
OR fasting OR "caloric restriction" OR "calorie restriction" OR "time restricted" OR "time-restricted" OR
vitamin* OR cholecalciferol OR cobalamin OR cyanocobalamin OR methylcobalamin OR folate OR "folic acid" OR
homocysteine OR creatine OR "coenzyme Q10" OR "coenzyme Q" OR ubiquinone OR ubiquinol OR CoQ10 OR
"nicotinamide riboside" OR probiotic* OR prebiotic* OR synbiotic* OR "omega-3" OR "omega 3" OR
docosahexaenoic OR DHA OR "fish oil" OR saffron OR crocin OR crocetin OR curcumin OR polyphenol* OR
flavonoid* OR "green tea" OR epigallocatechin OR EGCG OR "metabolic activator" OR "metabolic activators" OR
"nutritional ketosis" OR "ketone ester" OR "ketone esters") AND TS=(random* OR placebo* OR "controlled trial"
OR "controlled study" OR controlled OR "control group" OR "pilot trial" OR "pilot study" OR crossover OR
"cross-over") AND LA=(English)
```

The optional document-type limit DT=(Article OR Early Access) was not applied.

### *Cochrane Central Register of Controlled Trials (Cochrane Library, Search Manager; Issue 8 of 12, August 2026)*

```
#1 (parkinson*):ti,ab,kw

#2 (cognit* OR dementia OR "mild cognitive impairment" OR MCI OR neuropsycholog* OR memory OR "executive
function" OR "executive functions" OR attention OR MoCA OR "Montreal Cognitive Assessment" OR MMSE OR "mini-
mental" OR "mini mental"):ti,ab,kw

#3 (diet OR diets OR dietary OR nutrition* OR nutrient* OR nutraceutical* OR supplement* OR Mediterranean OR
"MIND diet" OR ketogenic OR ketone* OR ketosis OR "medium chain triglyceride" OR "medium chain triglycerides"
OR "medium-chain triglyceride" OR "medium-chain triglycerides" OR "low protein" OR "low-protein" OR "protein
redistribution" OR "protein restriction" OR fasting OR "caloric restriction" OR "calorie restriction" OR
"time restricted" OR "time-restricted" OR vitamin* OR cholecalciferol OR cobalamin OR cyanocobalamin OR
methylcobalamin OR folate OR "folic acid" OR homocysteine OR creatine OR "coenzyme Q10" OR "coenzyme Q" OR
ubiquinone OR ubiquinol OR CoQ10 OR "nicotinamide riboside" OR probiotic* OR prebiotic* OR synbiotic* OR
```

```
"omega-3" OR "omega 3" OR docosahexaenoic OR DHA OR "fish oil" OR saffron OR crocin OR crocetin OR curcumin
OR polyphenol* OR flavonoid* OR "green tea" OR epigallocatechin OR EGCG OR "metabolic activator" OR
"metabolic activators" OR "nutritional ketosis" OR "ketone ester" OR "ketone esters"):ti,ab,kw
#4 #1 AND #2 AND #3
```

The Trials count was read from line #4 (380 records in all content, of which 373 were Trials and 7 Cochrane Reviews; the Trials set was exported). Typed MeSH descriptor lines are not accepted by the Search Manager, so the strategy used free-text title, abstract and keyword terms only; the same text blocks were used in the other databases.

*Deduplication and pre-sorting*

Exports (PubMed format, RIS and CSV) were merged by script. Records were deduplicated by DOI, then PubMed identifier, then normalized title (letters and digits only, case-insensitive), in that order. Before manual screening, each unique record was pre-sorted by keyword rules applied to the title and abstract, in this sequence: (1) the 13 controlled studies already identified were tagged by DOI; (2) trial-registry entries and conference abstracts were tagged for exclusion; (3) records whose publication type or title indicated a review, editorial, protocol, chapter or erratum were tagged for exclusion; (4) records with animal or in vitro terms and no human-study terms were tagged for exclusion; (5) records whose title indicated Parkinson's disease risk, incidence or prodromal features without a cognition term were tagged for exclusion; (6) records with no dietary, nutritional or supplement term in the title or abstract were tagged for exclusion; (7) records with no cognition term were tagged for exclusion; (8) records with no controlled-design term were tagged as probable exclusions to be checked; the remainder were tagged as full-text candidates. All tags were suggestions only: every title was then screened by one author (N.Q.), who confirmed or reversed each tag, and the counts in Figure 1 are the author's decisions. The full screening workbook is available from the corresponding author.

Of the 13 controlled studies in the original submission, 11 were retrieved by the structured search. The NET-PD LS-1 report (JAMA 2015) and the KIM trial (J. Parkinsons Dis. 2026) were not retrieved, because neither abstract contains a cognition term; both had been identified by the original targeted retrieval and reference-list checking and are counted in Figure 1 as identified through other sources. The structured search identified ten additional randomized controlled studies with an objective cognitive outcome (DATATOP, primary report 1993 and cognitive brief communication 1994, tocopherol; Suzuki 2013, vitamin D; Wakade 2021 and the randomized phase of Chong 2021, niacin; Sun 2022, Zeng 2024 and Magistrelli 2024, probiotics; Bolner 2023, fermented papaya; Huang 2023, ginkgo leaf tablets; Shateri 2025, boswellic acids), which were added to Tables 2 and 3. Two non-randomized Pycnogenol registry studies with physician-rated cognitive scores (Cesarone 2020; Belcaro 2022), two randomized trials whose only cognitive measure was the PDQ-39 cognitive-impairment domain (Sheard 2014; Mehrabani 2023) are cited as context. Final adjudication on 2 October 2026 retained Chong’s randomized three-month phase because it reports arm-level Trail Making B data; a comparative p value is not required for eligibility. Its later uncontrolled phase remains context.

**Table S1. Domain-level appraisal of the 23 controlled studies**

Domains are informed by RoB 2 [Sterne et al., BMJ 2019;366:l4898] and supplemented by multiplicity, adherence/exposure and analysis denominators. This is a descriptive appraisal, not a formal RoB 2 assessment; no overall rating, score or GRADE classification is assigned. NR means not reported in the source material inspected, not proof that an item was absent from the original protocol. Rows marked † are the ten revision-stage additions. All ten were checked against full reports, including the Sun supplement and the 1994 DATATOP cognitive brief communication. DATATOP is one trial represented by the 1993 NEJM primary report (main reference 35; doi:10.1056/NEJM199301213280305) and the 1994 Neurology cognitive brief communication (main reference 36; doi:10.1212/WNL.44.9.1756). Trial design, conduct and adherence are taken from the primary report; cognitive scores, analysis populations, exclusions and multiplicity procedures are taken from the full brief communication. The cognitive analysis is distinct from the primary trial's intention-to-treat disability analysis. Registry identifiers are transcribed from reports, not confirmation of prospective registration or endpoint prespecification. Anamnart used a randomized no-additional-treatment comparator, not a placebo; Tosefsky and Bolner used crossover designs.

| Study | Randomization process | Allocation concealment | Blinding | Attrition and missing data | Registration and endpoint prespecification | Multiplicity | Adherence or exposure verification | n analyzed for cognition |
|---|---|---|---|---|---|---|---|---|
| Paknahad 2020 | Computer-generated (low) | NR (some concerns) | Participants and assessors reported blinded; dietary masking unclear (some concerns) | 12.5% attrition; completer analysis after post-randomization exclusion of non-adherent participants (high) | Registration NR; MoCA stated as study objective | Six MoCA subsections; no correction reported (some concerns) | 24-hour dietary recalls every 2 weeks, weekly telephone follow-up and monthly counseling; 5 excluded for non-adherence | 70 (35/35) |
| Krikorian 2019 | Random-number sequence (low) | NR (some concerns) | Diets not masked; assessor blinding NR (some concerns) | 22% attrition; completer analysis (high) | NCT00777010; cognition was the stated aim | Several cognitive composites; no correction reported (some concerns) | Weekly 3-day diaries; ketone checks at weeks 2/4/6. βHB 0.08 ± 0.04 → 0.31 ± 0.19 mM (low carbohydrate); 0.11 ± 0.06 → 0.08 ± 0.01 (control) | 14 (7/7) |
| Phillips 2018 | Online generator; calorie-stratified blocks of 2 (low) | Lead investigator generated and nurse witnessed sequence; concealment unclear (some concerns) | Diets not masked; patient-rated outcome (high) | 9/47 withdrew; ITT with regression imputation and sensitivity analyses (some concerns) | ACTRN12617000027314; Part I item exploratory | Multiple non-motor items (high for item-level inference) | Daily bedtime blood ketones; 8-week mean 1.15 ± 0.59 vs 0.16 ± 0.05 mmol/L; 16.3% recordings missing | 47 ITT (24 ketogenic/23 low-fat); 38 completed |

| Study | Randomization process | Allocation concealment | Blinding | Attrition and missing data | Registration and endpoint prespecification | Multiplicity | Adherence or exposure verification | n analyzed for cognition |
|---|---|---|---|---|---|---|---|---|
| Choi 2024 | Statistician-generated stratified blocks of 4 (low) | NR (some concerns) | Double-blind, inpatient controlled feeding (low) | All 16 completed randomized inpatient week; 1 subsequent extension withdrawal (low) | ClinicalTrials.gov NCT04584346; cognition exploratory | Multiple exploratory measures (some concerns) | Controlled inpatient meals; KD βHB 0.59 ± 0.25 mM by day 4. Home phase mean net carbohydrate 9.7% of energy | 16 for randomized week (7/9); 15 at week 3 |
| Tosefsky 2026 (KIM) | Random-order crossover; method NR (some concerns) | NR (some concerns) | Diets not masked; assessor blinding NR (some concerns) | 37% dropout; ITT and available-case analyses both reported (high) | ClinicalTrials.gov NCT05469997; cognition secondary | Secondary cognitive outcomes (some concerns) | Blood ketones measured; biochemical ketosis in about half (exposure incomplete) | 48 ITT; 33 both phases |
| Hiller 2018 | Pharmacy-generated stratified permuted blocks (low) | Independent VA pharmacy assigned coded treatment (low) | Double-blind placebo (low) | 12% attrition; completer analysis (some concerns) | NCT01119131; cognition secondary | Battery of tests, secondary (some concerns) | Serum 25-hydroxyvitamin D rise (low) | 51 completers; MoCA 50 (26/24); other tests 42–49 |
| NET-PD LS-1 2015 | Central randomization (low) | Central (low) | Double-blind placebo (low) | ITT; prespecified handling (low) | NCT00449865; SDMT part of global primary outcome; SCOPA-Cog secondary | Single global outcome (low) | Study-drug exposure 71% vs 79% of participant-years; 34% vs 26% stopped study drug | 1,741 randomized; 955 in 5-year interim ITT analysis; SCOPA-Cog 624 |
| QE3 2014 | Central randomization (low) | Central (low) | Double-blind placebo (low) | ITT (low) | NCT00740714; Symbol Digit secondary | Secondary outcome (low) | Medication logs and plasma CoQ10 at baseline and months 1/8/16; compliance reported similar between groups | 600 |
| Li 2015 | Computer-generated randomization (low) | NR (some concerns) | Blinding to allocation described; roles incompletely specified (some concerns) | Participant flow and missing-data handling NR; tables retain 38/37 (high) | Registration NR; MoCA primary | Single instrument at two time points (low) | Adherence measurement/result not reported in the full article | 75 (38/37) |

| Study | Randomization process | Allocation concealment | Blinding | Attrition and missing data | Registration and endpoint prespecification | Multiplicity | Adherence or exposure verification | n analyzed for cognition |
|---|---|---|---|---|---|---|---|---|
| Lam 2026 | Supplier-generated permuted blocks of 4 (low) | Coded identical sachets; all trial staff blinded (low) | Double-blind placebo (low) | 16% withdrew; ITT with all 61 (low) | NCT03968133; MoCA secondary | Multiple secondary outcomes; no correction reported (some concerns) | Returned-sachet counts among completers: 98.5 ± 3.6% probiotic vs 98.5 ± 2.8% placebo | 61 |
| Yulug 2025 | Method NR (some concerns) | NR (some concerns) | Double-blind placebo (low) | 10% attrition (some concerns) | NCT04044131; MoCA secondary | Multi-omics and subgroup analyses (high) | Protocol: weekly nurse telephone checks of compliance and adverse events; achieved adherence percentage not reported | 43 |
| Hajhashemy 2026 | Computer-generated stratified blocks (sex; Hoehn and Yahr 1–2 vs 3–4; size 4), independent allocator (low) | Opaque sealed envelopes held independently; coded A/B packages (low) | Matched tablets; participants, investigators, laboratory and statistical staff blinded (low) | 8/92 withdrew (3/5); ITT with EM imputation under a missing-at-random assumption; sensitivity analysis NR (some concerns) | IRCT20121216011763N61; published protocol cited; AMT secondary, CRP/oxidative-stress markers primary; prospective prespecification not independently verified | Multiple outcomes; no correction reported (high) | Telephone checks and tablet counts; achieved adherence percentage NR; serum crocin not measured | 92 randomized and analyzed (46/46); 84 completed (43/41) |
| Anamnart 2026 | Computer-generated blocked randomization (low) | NR (some concerns) | No placebo; no blinding (high) | No dropouts; all 60 analyzed (low) | TCTR20240708007; retrospectively registered; MMSE primary clinical outcome | Multiple clinical outcomes (some concerns) | Patient-reported adherence; 25(OH)D 25.03 ± 9.55 → 37.55 ± 10.58 ng/mL vs 27.72 ± 9.34 → 26.21 ± 8.80 | 60 PD participants (30/30); 60 healthy comparators not counted |
| Wakade 2021 † | Fixed pharmacy schedule; balanced blocks by Hoehn and Yahr stage | Sequestered pharmacy schedule; opaque pouches; code held until study end | Participants and researchers blinded; placebo controlled | 47 enrolled; 39 evaluated at 6 months; 31 at 12 months. ITT and per-protocol reported; LOCF explicitly described for motor outcome, not established for cognitive estimates | NCT03462680; cognition described as secondary in article; registry prespecification not independently checked | Bonferroni adjustment stated | Quantitative adherence NR | 39 at 6 months (18 niacin, 21 placebo); exact population underlying each cognitive estimate not separately specified |

| Study | Randomization process | Allocation concealment | Blinding | Attrition and missing data | Registration and endpoint prespecification | Multiplicity | Adherence or exposure verification | n analyzed for cognition |
|---|---|---|---|---|---|---|---|---|
| Sun 2022 † | 100 allocated 1:1 (50 per arm); sequence-generation method NR | NR | Double-blind stated; matching appearance and taste; roles not fully specified | 18/100 excluded during intervention: 2/50 probiotic, 16/50 placebo. Clinical analyses: 82 (48/34); omics: 74 (45/29). Differential exclusions are a major concern | ChiCTR1800016977; MMSE among clinical indexes; separate cognitive endpoint hierarchy NR; registry prespecification not independently checked | Multiple outcomes; no correction reported for MMSE comparisons | Strain engraftment shown; quantitative adherence NR | Clinical analysis set 82 (48 probiotic, 34 placebo); 74 is the omics set, not the clinical set |
| Huang 2023 † | Random-number table; 80 allocated 40/40 | NR | Roles and procedures NR; do not infer an open design | Attrition and missing-data handling NR; tables retain 40 per arm | Registration NR; MMSE and MoCA are study objectives, but formal primary/secondary designation and prospective prespecification are not established | Multiple outcomes; no adjustment reported | NR | Outcome-specific analyzed n NR; 80 randomized (40/40) |
| Shateri 2025 † | Computer-generated block randomization (block size 4) managed by a coordinator not involved in data collection or evaluation (low) | Sealed opaque sequentially numbered envelopes (low) | Identical capsules; patients, researchers and assessing neurologists blinded (low) | 14 of 58 withdrew (7 per arm, including 3 deaths); six-month MoCA available for 12 of 29 vs 15 of 29 (high) | IRCT20170315033086N4, registered retrospectively on 12 April 2019 with inflammatory cytokines as the only registered outcome; the report names UPDRS III the primary clinical outcome; MoCA secondary, not registered | UPDRS, MoCA and 12 cytokines; no adjustment (some concerns) | Weekly pill counts, self-report and caregiver logs; per-protocol exclusion if >15% of doses missed; compliance figures NR | 12 boswellic acids, 15 placebo (six-month MoCA) |
| Suzuki 2013 † | Central computerized allocation; permuted blocks of four | Central allocation | Patients, families and evaluating doctors blinded; identical tablets | 10/114 withdrew (7/56 vitamin D, 3/58 placebo). Authors' ITT set 112 (55/57), excluding one lost participant per arm; cognitive denominators require qualification | UMIN000001841; MMSE among article-defined primary measures; registry prespecification not independently checked | Monte Carlo permutation procedures reported for multiple testing | Bottle checks; compliance 49/55 (89.1%) vs 55/57 (96.5%); serum 25(OH)D rise | Table 2 headers: 55/57; MMSE not-worsened counts/percentages imply 49/55. Exact n for mean MMSE change not explicit |

| Study | Randomization process | Allocation concealment | Blinding | Attrition and missing data | Registration and endpoint prespecification | Multiplicity | Adherence or exposure verification | n analyzed for cognition |
|---|---|---|---|---|---|---|---|---|
| DATATOP 1993/1994 † | Random assignment, 2 × 2 factorial, balanced within each investigator's center; sequence generation method NR (some concerns) | Identical-appearing active and placebo preparations; investigators and participants unaware; central procedure NR (some concerns) | Double-blind; identical-appearing preparations; cognitive tests administered by trained personnel using uniform scoring (low) | 90/800 excluded before 6 months owing to endpoint or termination; 710 in cognitive table. Primary disability endpoint used ITT; cognition did not (some concerns) | Pre-registration era; cognition secondary; prospective six-monthly assessments; annualized-change analysis described | Bonferroni $p < 0.0033$ for 15 cognitive variables; ANCOVA adjusted for age, gender and center | 97.9–99.5% of dispensed doses taken; serum tocopherol >2.0 mg/dL in 78–90% assigned tocopherol vs 0–19% not assigned (primary report) | 799 baseline; 710 longitudinal: 174 placebo, 174 tocopherol, 187 deprenyl, 175 combination (published table) |
| Zeng 2024 † | Python-generated random-number table; 1:1 allocation (23/23) | Concealed random allocation stated | Similar products; dispensing nurse, participants, follow-up/evaluation, data-entry and statistical personnel blinded | 4/46 exited (2 per arm); 42 completed (21/21). ITT 46 and per-protocol 42 reported; imputation method NR | ChiCTR2000035122; MMSE listed in Methods; formal primary/secondary designation NR; registry prespecification not independently checked | Multiple outcomes; no correction reported | Taking <20% of required dose was an exit criterion; quantitative adherence NR | ITT 46 (23/23); per-protocol 42 (21/21) |
| Magistrelli 2024 † | Blind randomization stated; sequence method NR | NR | Blinding stated; maltodextrin placebo; roles not fully specified | 2/40 dropped out; one supplied clinical data. Exact MoCA analysis denominator not explicit; do not equate completion with cognitive analysis | ClinicalTrials.gov registration in November 2021 stated without identifier; formal cognitive endpoint hierarchy NR | Multiple outcomes; no adjustment reported; cognitive results are within-group tests | Quantitative adherence NR; product supplied by manufacturer | Exact MoCA n NR; 40 enrolled, 38 completed, with clinical data retained from one dropout |
| Bolner 2023 † | Randomized 1:1; 19 active-first, 20 placebo-first; sequence method NR | NR | Double-blind stated; roles and procedures insufficiently detailed | Attrition, missing-data handling and completers per period NR | Registration NR; MoCA and FAB listed in Methods; formal hierarchy and prospective prespecification not established | Nine scales; t and Wilcoxon tests; pooled treatment periods; no washout reported or period/carryover model | Quantitative adherence NR | Outcome-specific n NR; 39 randomized (19/20 by sequence) |

| Study | Randomization process | Allocation concealment | Blinding | Attrition and missing data | Registration and endpoint prespecification | Multiplicity | Adherence or exposure verification | n analyzed for cognition |
|---|---|---|---|---|---|---|---|---|
| Chong 2021 † | Random allocation blocked by Hoehn and Yahr severity; sequence-generation details NR | NR | Double-blind stated; specific roles and matching procedures NR | 47 randomized (16/16/15, Figure 1); one dropout from the 100-mg arm; 46 completed 3 months (16/15/15). Dropout reason and cognitive missing-data method NR | Registration NR; UPDRS III primary; cognitive tests listed, but prospective cognitive hierarchy not independently established | No three-month cognitive comparative analysis or multiplicity procedure reported; later analyses not applied to randomized phase | Quantitative adherence NR | Cognitive-specific n NR; 47 randomized, 46 completed. Figure 1: allocated 16/16/15; completed 16/15/15 (placebo/100 mg/250 mg) |

Abbreviations: AMT, Abbreviated Mental Test; ITT, intention-to-treat; EM, expectation–maximization; KIM, Mediterranean–Ketogenic Interventions in Parkinson's disease trial; MMSE, Mini-Mental State Examination; MoCA, Montreal Cognitive Assessment; NR, not reported.

**Table S2. Cognitive Score Sequences, Adherence, Attrition and Adverse Events**

| Study | Instrument | Intervention: baseline, follow-up or change (format specified) | Control: baseline, follow-up or change (format specified) | Between-group result | Adherence or exposure | Attrition | Adverse events (as extracted) |
|---|---|---|---|---|---|---|---|
| Paknahad 2020 | Persian MoCA | 19.18 ± 5.79 → 20.79 ± 5.12 (change +1.61 ± 2.25) | 19.67 ± 5.76 → 19.06 ± 5.83 (change −0.61 ± 1.11) | Difference in change 2.22; endpoint difference 1.73 (both unadjusted; calculated by the review authors); reported GLM $p = 0.001$; CI not reported | 24-hour dietary recalls every 2 weeks, weekly telephone follow-up and monthly counseling; 5 excluded for non-adherence | 10/80 (12.5%): 5 diet non-adherence; 5 controls unwilling to continue | Adverse-event results not reported in the full article |
| Krikorian 2019 | Lexical access and memory composites | Composite arm means/SDs not tabulated; representative COWA and paired-associate results graphed in Figure 2 | Composite arm means/SDs not tabulated; representative task results graphed in Figure 2 | Baseline-adjusted $p = 0.02$ ($f = 0.76$) and $p = 0.01$ ($f = 0.87$); CI not reported | Weekly 3-day diaries; ketone checks at weeks 2/4/6. βHB 0.08 ± 0.04 → 0.31 ± 0.19 mM (low carbohydrate); 0.11 ± 0.06 → 0.08 ± 0.01 (control) | 4/18 withdrew: 3 longstanding medical conditions, 1 perceived fatigue; 7/7 completers | One withdrawal for perceived fatigue; 3 for pre-existing conditions. Low-carbohydrate weight 89.9 ± 12.1 → 83.4 ± 12.5 kg; no systematic AE table |

| Study | Instrument | Intervention: baseline, follow-up or change (format specified) | Control: baseline, follow-up or change (format specified) | Between-group result | Adherence or exposure | Attrition | Adverse events (as extracted) |
|---|---|---|---|---|---|---|---|
| Phillips 2018 | MDS-UPDRS Part I cognitive-impairment item | Cognitive-item arm means/SDs not reported in article or appendix; Part I total 11.15 ± 4.15 → 6.57 ± 4.09 (not an isolated cognitive score) | Cognitive-item arm means/SDs not reported; Part I total 8.96 ± 4.34 → 7.96 ± 6.56 | Published cognitive-item change difference −0.27 points (ketogenic minus low-fat); item-specific CI and p not given in main text. Part I total $p < 0.001$ must not be assigned to the item | Daily bedtime blood ketones; 8-week mean 1.15 ± 0.59 vs 0.16 ± 0.05 mmol/L; 16.3% recordings missing | 9/47 withdrew (6 ketogenic, 3 low-fat); 38 completed; ITT 47 with regression imputation | Weeks 1–4 tremor/rigidity: 12/24 ketogenic vs 3/23 low-fat; 2 ketogenic withdrawals. Hunger: 4/24 vs 5/23. Mean weight loss 4.37 vs 4.87 kg |
| Choi 2024 | 3-back, Stroop, reaction time | Day 1 → 7: 3-back correct 68.57 ± 14.92 → 75.71 ± 23.35%; reaction time 1123.01 ± 236.73 → 958.06 ± 332.66 ms. Full controlled-phase battery: Table S5 | Day 1 → 7: 3-back correct 63.89 ± 22.19 → 78.89 ± 9.28%; reaction time 1119.08 ± 347.56 → 822.34 ± 251.52 ms. Full battery: Table S5 | Similar improvement reported in both groups; comparative cognitive estimates/CIs not reported. Week 3 was an uncontrolled ketogenic extension | Controlled inpatient meals; KD βHB 0.59 ± 0.25 mM by day 4. Home phase mean net carbohydrate 9.7% of energy | 16 completed the randomized week (7/9); 1 standard-diet participant withdrew in the subsequent home ketogenic phase; 15 completed week 3 | Inpatient indigestion/reflux 2/7 vs 1/9; fatigue 1/7 vs 1/9. Home phase: fatigue 5/15; one DVT/pulmonary embolus judged unlikely related |
| Tosefsky 2026 (KIM) | MoCA; PD-CRS | MeDi-KD baseline → post (n = 43/39): MoCA 27.4 ± 2.2 → 27.31 ± 2.3; PD-CRS 88 ± 23 → 86 ± 21 | MeDi-MCT baseline → post (n = 46/41): MoCA 27.2 ± 2.3 → 27.6 ± 2.3; PD-CRS 88 ± 18 → 88 ± 19 | No between-intervention MoCA difference. KD PD-CRS within-phase decrease $p = 0.024$ in available cases, not reproduced in ITT; no comparative estimate/CI tabulated | Home ketone monitoring and dietitian contacts; fasting βHB >0.5 mM in 50% after KD vs 3% after MCT | 52 enrolled; 48 started; 41 completed ≥1 phase, 33 both. Table 4 uses phase-specific available samples | Table 3 lists 8 KD and 10 MCT events; gastrointestinal, hypotensive and motor symptoms. No intervention-related serious AE; 2 unrelated hospitalizations during MCT |
| Hiller 2018 | MoCA; neuropsychological battery | MoCA baseline 23.88 ± 2.79; change +0.9615 ± 2.20 (n = 26). Other cognitive changes: Table S4 | MoCA baseline 24.83 ± 3.13; change +1.042 ± 2.51 (n = 24). Other cognitive changes: Table S4 | MoCA change comparison $p = 0.905$; no cognitive battery comparison significant. Published per-arm CIs and other test results in Table S4 | Serum 25(OH)D increased from about 30 to 61 ng/mL in the supplemented group; biochemical exposure verified | 7/58 (12.1%) withdrew; 51 completers (27/24); MoCA n = 50; other tests have varying n | Table 4 lists 12 vs 20 adverse events; no serious AEs or hypercalcemia; no abnormal calcium, phosphate or creatinine values |

| Study | Instrument | Intervention: baseline, follow-up or change (format specified) | Control: baseline, follow-up or change (format specified) | Between-group result | Adherence or exposure | Attrition | Adverse events (as extracted) |
|---|---|---|---|---|---|---|---|
| NET-PD LS-1 2015 | SDMT (primary global outcome component); SCOPA-Cog (secondary) | SDMT baseline 44.4 ± 11.8; 5-year worsening-coded change +4.9 ± 17.7 (n = 477, imputed). SCOPA-Cog change −1.9 ± 5.4 (n = 309) | SDMT baseline 44.5 ± 11.6; worsening-coded change +4.5 ± 16.8 (n = 478, imputed). SCOPA-Cog change −2.0 ± 4.9 (n = 315) | Placebo minus creatine: SDMT worsening-coded change −0.4 (95% CI −2.7 to 1.8); SCOPA-Cog change −0.1 (−0.9 to 0.7). Neither favored creatine | Study-drug exposure 71% vs 79% of participant-years; 34% vs 26% stopped study drug | At July 2013 cutoff: 668/874 creatine and 669/867 placebo retained; 955 eligible for 5-year interim efficacy analysis | No AE/serious-AE body-system difference at prespecified $p < 0.01$; creatinine increased initially. Deaths 44 vs 36; 41 vs 8 stopped drug for protocol renal thresholds |
| QE3 2014 | Symbol Digit Modalities Test | Baseline SDMT (SD): 44.0 (16.7) at 1,200 mg; 43.0 (16.4) at 2,400 mg. Repeated-visit adjusted changes (SE): −0.49 (1.39); −3.36 (1.40) | Baseline SDMT 43.6 (SD 17.0); repeated-visit adjusted change −3.02 (SE 1.48) | No significant between-group SDMT difference; dose-specific contrasts/CIs not tabulated. Changes adjusted for baseline and enrolling site | Medication logs and plasma CoQ10 at baseline and months 1/8/16; compliance reported similar between groups | 65/600 premature withdrawals: 19/201 at 1,200 mg; 17/196 at 2,400 mg; 29/203 placebo | 73.5% reported ≥1 AE; no overall group difference. 41 serious events in 33 participants; 1 GI bleed possibly treatment-related. AE/intolerance withdrawals 10/397 vs 4/203 |
| Li 2015 | MoCA | 20.15 ± 3.11 → 19.52 ± 2.74 (12 mo) → 18.55 ± 4.11 (18 mo); change −1.60 | 19.63 ± 4.12 → 16.33 ± 3.37 (12 mo) → 13.33 ± 3.58 (18 mo); change −6.30 | Endpoint difference 5.22 at 18 months (unadjusted; calculated by the review authors); reported $p < 0.05$ at 12 months, $p < 0.01$ at 18 months; CI not reported | Adherence measurement/result not reported in the full article | Withdrawal flow and missing-data handling not reported; outcome tables retain 38/37 | Two controls developed dementia by 18 months; systematic adverse-event results not reported |
| Lam 2026 | MoCA | ITT means (SE): 27.4 (0.4) → 28.7 (0.4); adjusted change +1.3 (95% CI 0.4–2.2) | ITT means (SE): 27.3 (0.3) → 27.6 (0.4); adjusted change +0.3 (95% CI −0.5 to 1.2) | Adjusted mean difference +1.1 (95% CI 0.04–2.1; p = 0.043) | Returned-sachet counts among completers: 98.5 ± 3.6% probiotic vs 98.5 ± 2.8% placebo | 10/61 withdrew: 3/30 probiotic, 7/31 placebo; all 61 in ITT analysis | Possibly/probably/definitely related AEs in 10/30 vs 12/31 (p = 0.79), mainly gastrointestinal; no serious AEs |
| Yulug 2025 | MoCA | MoCA mean ± SD at days 0/28/84: 15.66 ± 4.58 / 17.66 ± 5.63 / 18.89 ± 5.83; n = 32/32/28 (calculated from published Dataset 1) | MoCA at days 0/28/84: 17.69 ± 5.19 / 19.75 ± 5.93 / 19.67 ± 6.63; n = 16/16/15 (calculated from Dataset 1) | Between-group p = 0.233/0.219/0.564 at days 0/28/84; day-84 log2 fold-change contrast −0.058. Raw-score adjusted contrast/CI not reported; within-arm changes do not establish benefit | Protocol: weekly nurse telephone checks of compliance and adverse events; achieved adherence percentage not reported | 5/48 withdrew before day 84 because of COVID-19 lockdown: 4 active and 1 placebo | No severe AEs. Active: moderate liver-enzyme increase (relationship unknown); placebo: moderate diarrhea and mild peripheral edema |

| Study | Instrument | Intervention: baseline, follow-up or change (format specified) | Control: baseline, follow-up or change (format specified) | Between-group result | Adherence or exposure | Attrition | Adverse events (as extracted) |
|---|---|---|---|---|---|---|---|
| Hajhashemy 2026 | AMT (secondary); patient-rated PDQ-39 cognitive domain separately in S6 | AMT 8.10 (0.31) → 8.95 (0.21); change +0.84 (0.22); within-arm $p < 0.001$. Mean (SE), ITT n = 46 | AMT 8.10 (0.30) → 8.17 (0.31); change +0.06 (0.30); within-arm p = 0.83. Mean (SE), ITT n = 46 | Crude +0.78 (95% CI 0.03–1.53); baseline-adjusted ANCOVA +0.78 (95% CI 0.16–1.40), p = 0.01; ITT n = 92 | Telephone checks and tablet counts; achieved adherence percentage NR; serum crocin not measured | 8/92 withdrew: saffron 3 (lost 1, discontinued 1, changed medicines 1); placebo 5 (lost 2, discontinued 1, changed medicines 1, daily saffron tea 1). ITT with EM imputation | Weekly telephone ascertainment; no side effects or adverse-event-related withdrawals reported |
| Anamnart 2026 | Thai MMSE | Thai MMSE 25.83 ± 3.35 → 27.20 ± 2.48; change +1.37 ± 1.92 | Thai MMSE 25.83 ± 3.29 → 26.77 ± 2.90; change +0.93 ± 1.86 | Difference in change +0.43 (95% CI −0.54 to 1.41; p = 0.378); endpoint difference +0.43 (−0.96 to 1.83; p = 0.536) | Patient-reported adherence; 25(OH)D 25.03 ± 9.55 → 37.55 ± 10.58 ng/mL vs 27.72 ± 9.34 → 26.21 ± 8.80 | 0/60 dropped out; 30/30 analyzed; healthy comparators excluded from the intervention denominator | No participant reported vitamin D3 side effects |
| Wakade 2021 † | Trail Making Test B−A (s); Stroop third trial | Mean ± SD, baseline → 6 months: TMT B−A 79.42 ± 62.46 → 74 ± 54.7; Stroop 6.18 ± 7.92 → 8.4 ± 5.6 | Mean ± SD, baseline → 6 months: TMT B−A 73.38 ± 57.11 → 76.4 ± 63.1; Stroop 8.13 ± 6.64 → 8.7 ± 6.9 | Reported comparative estimates (95% CI): TMT B−A −6.04 (−54.93 to 42.85); Stroop 1.96 (−4.16 to 8.08). Neither significant; no recalculation of the source's contrast | Quantitative adherence NR | 8/47 before 6 months; 8 further before 12 months | Full-study report: flushing in 2 (one per arm, both discontinued); unrelated injuries in 2; leg cramps in 1 |
| Sun 2022 † | MMSE | Mean ± SD: baseline 25.26 ± 4.01; 1 month 26.33 ± 3.69; 3 months 26.63 ± 3.75 | Mean ± SD: baseline 25.47 ± 4.37; 1 month 25.71 ± 4.32; 3 months 25.88 ± 4.41 | Supplementary Table 5: between-group p = 0.71 at baseline, 0.57 at 1 month, 0.44 at 3 months. No significant difference; change-based contrast and CI NR | Strain engraftment shown; quantitative adherence NR | 18/100 during intervention: 2/50 probiotic, 16/50 placebo; clinical set 48/34. A further 8 lacked complete omics samples | No additional adverse events of any severity versus placebo reported; numerical event counts NR |
| Huang 2023 † | MMSE and MoCA | Scores displayed graphically; exact means/SD not numerically tabulated. No values imputed or digitized | Scores displayed graphically; exact means/SD not numerically tabulated | Figure 1: post-treatment comparison favors combination for both instruments ($p < 0.05$); numeric effect estimates and CI NR | NR | NR; tables retain 40 per arm | Table 4: 7/40 (17.5%) with combination vs 12/40 (30%) with control; p = 0.189 |

| Study | Instrument | Intervention: baseline, follow-up or change (format specified) | Control: baseline, follow-up or change (format specified) | Between-group result | Adherence or exposure | Attrition | Adverse events (as extracted) |
|---|---|---|---|---|---|---|---|
| Shateri 2025 † | MoCA | 22.42 ± 4.93 → 23.67 ± 3.82 at 6 months (paired p = 0.1872); n = 12 at 6 months | 21.00 ± 6.92 → 21.40 ± 7.33 at 6 months (paired p = 0.8646); n = 15 at 6 months | Between-group contrast NR (baseline p = 0.734 only) | Weekly pill counts, self-report, caregiver logs; compliance figures NR | 14 of 58 (24%); 6-month MoCA in 27 of 58 | No severe adverse events reported; laboratory parameters unchanged; three deaths listed among withdrawals (2 vs 1) |
| Suzuki 2013 † | Japanese MMSE | Baseline median 28 (IQR 26–30); 12-month change −0.33 ± 2.16 (mean ± SD) | Baseline median 28 (IQR 26–30); 12-month change +0.27 ± 1.74 (mean ± SD) | Change p = 0.12 (permutation test). Not worsened: 31 (63.3%) vs 43 (78.2%); RR 0.81 (95% CI 0.63–1.04); RD −0.15 (−0.32 to 0.02). Denominators implied by percentages: 49/55, not header 55/57 | Compliance 89.1% vs 96.5%; serum 25(OH)D 22.5 → 41.7 vs 21.1 → 21.4 ng/mL | 7/56 vitamin D vs 3/58 placebo withdrew; authors' ITT 55/57 | One cerebral infarction in vitamin D arm; no vitamin D-arm hypercalcemia. One placebo participant had calcium 11.0 mg/dL |
| DATATOP 1993/1994 † | Seven cognitive instruments; 11 published score rows (Table S3) | Tocopherol / combination annualized change (mean ± SD): MMSE −0.1 ± 1.4 / +0.4 ± 1.0; Symbol Digit −0.7 ± 9.0 / +0.9 ± 6.7. Full battery: Table S3 | Placebo / deprenyl annualized change (mean ± SD): MMSE +0.3 ± 1.1 / 0.0 ± 1.1; Symbol Digit −0.9 ± 8.0 / +0.3 ± 7.3. Full battery: Table S3 | No significant tocopherol or deprenyl effect after Bonferroni correction; adjusted treatment estimates, 95% CIs and exact tocopherol p values not reported | 97.9–99.5% of doses taken; serum tocopherol consistent with assignment (primary report) | 90 excluded from cognitive change before 6 months; 710 tabulated. Separately, 57/800 withdrew before the primary disability endpoint | Adverse symptoms infrequent and uniform across groups; no deaths during this phase (primary report) |
| Zeng 2024 † | MMSE (Table 2); Δ denotes change from baseline, not endpoint score | ITT (n = 23): baseline 28.87 ± 1.63; Δ6 weeks +0.09 ± 0.73; Δ12 weeks +0.39 ± 1.58. Per-protocol (n = 21): baseline 29.00 ± 1.58; Δ6 weeks +0.10 ± 0.77; Δ12 weeks +0.43 ± 1.21 | ITT (n = 23): baseline 28.91 ± 2.17; Δ6 weeks +0.39 ± 1.30; Δ12 weeks +0.57 ± 1.40. Per-protocol (n = 21): baseline 28.86 ± 2.26; Δ6 weeks +0.43 ± 1.363; Δ12 weeks +0.62 ± 1.47 | ITT between-group p: baseline 0.939; Δ6 weeks 0.335; Δ12 weeks 0.650. Per-protocol: 0.787, 0.558, 0.940, respectively. All values reproduced as printed; CI NR | <20% of required dose was an exit criterion; quantitative adherence NR | 2/23 per arm exited; ITT 46, per-protocol 42. Supplement: medication change and adverse event; placebo: other trial and travel restriction | Supplement: eczema (1, resolved without withdrawal), hypotension/dizziness (1, withdrew). Placebo: bloating (2). No serious adverse event reported |
| Magistrelli 2024 † | MoCA | Mean ± SD: 27.47 ± 1.81 → 28.05 ± 1.68; within-group p = 0.078 | Mean ± SD: 26.30 ± 2.77 → 25.70 ± 2.98; within-group p = 0.065 | Baseline between-group p = 0.222; follow-up or change-based between-group contrast NR | Quantitative adherence NR | 2/40 dropped out; one supplied clinical data; exact MoCA denominator NR | Systematic adverse-event counts NR; COVID-related dropout not attributed to supplement |
| Bolner 2023 † | MoCA; FAB | Score means/SD NR. Pooled active periods: MoCA p = 0.015 (t) / 0.025 (Wilcoxon); FAB p = 0.453 / 0.82 | Score means/SD NR. Pooled placebo periods: MoCA p = 0.734 (t) / 0.704 (Wilcoxon); FAB p = 0.562 / 0.779 | Between-treatment contrast NR; within-period significance is not a treatment effect | NR | NR; analyzed n per period not specified | NR |

| Study | Instrument | Intervention: baseline, follow-up or change (format specified) | Control: baseline, follow-up or change (format specified) | Between-group result | Adherence or exposure | Attrition | Adverse events (as extracted) |
|---|---|---|---|---|---|---|---|
| Chong 2021 † | Trail Making Test B, first trial (seconds); randomized phase, Table 2 | Mean ± SD, baseline → 3 months: niacin 100 mg 109.3 ± 138.6 → 104.7 ± 123.5; niacin 250 mg 95.3 ± 70.8 → 92.5 ± 72.8 | Placebo: 73.6 ± 30.5 → 69.7 ± 44.9 (mean ± SD) | Three-month between-arm estimate, test and CI NR. Table 2 p = 0.043 relates to later baseline-to-12-month analysis, not a randomized treatment contrast | Quantitative adherence NR | 46/47 completed 3 months; one dropout in the 100-mg arm (Figure 1); reason and cognitive-specific n NR. Later withdrawals excluded from this phase | Run-in flushing reported in 80% of 100-mg group and 5% of 250-mg group, lasting 30–45 min; placebo rate NR. Percentages as printed, not converted to inferred event counts |

Values are mean ± SD, mean (SE), median (IQR), or estimate (95% CI), as labeled. Sources are cited in Table 2 of the main manuscript. NR denotes information not reported in the inspected source. Saffron results are mean (SE) from the intention-to-treat analysis with expectation–maximization imputation; its AMT and patient-rated PDQ-39 cognitive-domain results are given separately in Table S6. Yulug means and sample SDs were calculated from published Supplementary Dataset 1 using available observations without imputation; published inferential tests were retained. NET-PD SDMT changes are worsening-coded (positive means decline), whereas baseline SDMT and SCOPA-Cog retain their higher-is-better direction. QE3 adjusted changes summarize repeated visits, not a single endpoint. For Zeng, 6- and 12-week values are changes from baseline; no endpoint SDs were imputed. Its placebo per-protocol 6-week SD of 1.363 and p values are retained as printed. Sun scores and flow come from supplementary Table 5 and Figure 1. Huang graph heights were not converted to exact numerical values. Suzuki denominators differ between mean-change and responder analyses and are labeled accordingly. DATATOP represents one trial with two reports; all published cognitive score rows are in Table S3. Chong uses randomized-phase three-month data only: allocation 16/16/15 and completion 16/15/15; later uncontrolled p values are not comparative treatment effects. Hiller and Choi battery results are in Tables S4 and S5. ITT, intention-to-treat; EM, expectation–maximization; PP, per-protocol; CI, confidence interval; SD, standard deviation; SE, standard error; IQR, interquartile range.

## Table S3. DATATOP Cognitive Scores and Annualized Changes

Values are mean ± SD. The baseline column describes the total cohort; the four treatment columns describe annualized change in raw-score points per year over a mean follow-up of 14 ± 6 months. Treatment-arm baseline means and endpoint means were not tabulated.

| Cognitive measure | Baseline total (n = 799) | Placebo (n = 174) | Tocopherol (n = 174) | Deprenyl (n = 187) | Combination (n = 175) |
|---|---|---|---|---|---|
| Digit Span forward | 8.9 ± 2.5 | −0.2 ± 2.2 | −0.1 ± 2.6 | 0.0 ± 2.0 | 0.1 ± 1.6 |
| Digit Span backward | 6.8 ± 2.4 | −0.1 ± 2.1 | −0.1 ± 2.4 | 0.1 ± 1.7 | 0.0 ± 1.7 |
| Symbol Digit | 38.8 ± 11.1 | −0.9 ± 8.0 | −0.7 ± 9.0 | 0.3 ± 7.3 | 0.9 ± 6.7 |
| Selective Reminding: total recall | 44.2 ± 10.2 | 0.7 ± 10.0 | 1.0 ± 9.0 | 0.7 ± 7.0 | 1.7 ± 7.2 |
| Selective Reminding: long-term recall | 32.3 ± 14.6 | 1.5 ± 12.4 | 0.7 ± 13.1 | 0.4 ± 10.5 | 2.3 ± 10.9 |
| Selective Reminding: delayed recall | 7.0 ± 2.7 | −0.2 ± 2.6 | −0.3 ± 2.7 | −0.4 ± 2.3 | −0.2 ± 2.1 |
| Odd Man Out: trials 1 + 3 | 17.8 ± 2.6 | 0.0 ± 2.6 | 0.1 ± 3.3 | 0.3 ± 3.2 | 0.0 ± 2.6 |
| Odd Man Out: trials 2 + 4 | 17.3 ± 3.2 | 0.9 ± 3.2 | 0.6 ± 3.5 | 0.4 ± 2.8 | 0.4 ± 2.9 |
| Spot-the-Dot | 12.8 ± 1.4 | −0.2 ± 1.7 | −0.3 ± 1.9 | −0.2 ± 1.4 | −0.3 ± 1.3 |

| Cognitive measure | Baseline total (n = 799) | Placebo (n = 174) | Tocopherol (n = 174) | Deprenyl (n = 187) | Combination (n = 175) |
|---|---|---|---|---|---|
| Verbal fluency | 27.5 ± 9.7 | 5.0 ± 7.9 | 4.5 ± 9.0 | 4.6 ± 7.8 | 5.2 ± 6.8 |
| Mini-Mental State Examination | 28.8 ± 1.4 | 0.3 ± 1.1 | −0.1 ± 1.4 | 0.0 ± 1.1 | 0.4 ± 1.0 |

Source: Kieburtz et al. Neurology 1994;44:1756–1759, Table, p. 1757 (main reference 36; doi:10.1212/WNL.44.9.1756). Arm sizes are the longitudinal sample sizes printed in that table and sum to 710. The report states 800 randomized, 799 baseline cognitive assessments and 90 exclusions from annualized-change analyses because the disability endpoint or premature termination occurred before the six-month visit. These denominators are retained as published. Positive annualized values indicate score increases.

Treatment effects were assessed by ANCOVA adjusted for age, gender and enrolling center, including deprenyl, tocopherol and their interaction. The report specified Bonferroni correction for 15 cognitive variables (two-sided nominal $p < 0.0033$); neither treatment significantly affected cognitive change after correction. The table publishes 11 score rows. Adjusted between-group estimates, their 95% confidence intervals and exact tocopherol p values were not reported. The nominal deprenyl trend on Symbol Digit ($p < 0.01$) did not meet the corrected threshold. These arm-level means are not adjusted treatment effects.

## Table S4. Hiller 2018 Cognitive Change Scores

| Measure | n | Vitamin D change (SD) | Vitamin D 95% CI | n | Placebo change (SD) | Placebo 95% CI | Between-group p |
|---|---|---|---|---|---|---|---|
| MoCA | 26 | 0.9615 (2.2) | 0.116, 1.807 | 24 | 1.042 (2.51) | 0.038, 2.046 | 0.905 |
| Judgment of Line Orientation | 26 | 0.1538 (2.461) | -0.792, 1.1 | 23 | 0.1739 (2.037) | -0.659, 1.006 | 0.975 |
| Vegetable Fluency | 25 | 0.44 (3.203) | -0.816, 1.696 | 23 | -1.13 (4.224) | -2.856, 0.596 | 0.157 |
| Digit Span—Forwards | 25 | 0.12 (1.922) | -0.633, 0.873 | 23 | 0.5217 (1.163) | 0.046, 0.997 | 0.382 |
| Digit Span—Backwards | 25 | 0.08 (2.019) | -0.711, 0.871 | 23 | 0.08696 (1.975) | -0.72, 0.894 | 0.99 |
| Boston Naming Task | 24 | 0.5417 (1.382) | -0.011, 1.095 | 23 | 0.4783 (1.238) | -0.028, 0.984 | 0.869 |
| Animal Fluency | 24 | 0.7917 (3.989) | -0.804, 2.388 | 23 | -1.217 (5.018) | -3.268, 0.834 | 0.137 |
| Letter-Number Sequence | 24 | 0.25 (1.984) | -0.544, 1.044 | 23 | -0.08696 (2.521) | -1.117, 0.943 | 0.614 |
| Digit Symbol Test | 24 | -0.6667 (5.947) | -3.046, 1.713 | 21 | 2 (10.56) | -2.517, 6.517 | 0.314 |
| Trails A (sec) | 26 | 0.4615 (19.65) | -7.092, 8.015 | 23 | -0.5652 (20.05) | -8.759, 7.629 | 0.857 |
| Trails B (sec) | 23 | 11.65 (49.03) | -8.388, 31.688 | 21 | -17.05 (77.31) | -50.116, 16.016 | 0.155 |
| Trails B-A | 23 | 12.13 (49.83) | -8.235, 32.495 | 21 | -17.33 (74.7) | -49.28, 14.62 | 0.137 |
| Immediate Logical Memory Score | 21 | 1 (4.359) | -0.864, 2.864 | 21 | 1.286 (3.901) | -0.382, 2.954 | 0.824 |
| Delayed Logical Memory Score | 22 | 1.273 (3.731) | -0.286, 2.832 | 21 | 0.619 (4.522) | -1.315, 2.553 | 0.609 |

Source: Hiller et al., PLoS ONE 2018;13:e0203637, Table 3 (main reference 87). Changes are baseline to 16 weeks, mean (SD), in the source units; Trails A and B are seconds. The source reports unadjusted two-sample t-test p values. Outcome-specific denominators vary. No cognitive comparison was significant; these are arm-level changes, not adjusted treatment effects.

## Table S5. Choi 2024 Cognitive Scores During the Randomized Inpatient Phase

| Measure | Ketogenic day 1 | Ketogenic day 7 | Standard day 1 | Standard day 7 |
|---|---|---|---|---|
| Simple reaction time (ms) | 365.53 (46.47) | 432.93 (181.67) | 393.09 (77.53) | 396.32 (55.38) |
| Choice reaction time (ms) | 762.47 (368.35) | 762.39 (408.12) | 847.2 (433.13) | 630.11 (75.86) |
| Stroop congruent time (ms) | 1281.67 (262.85) | 1049.09 (159.57) | 1253.11 (218.47) | 955.1 (114.89) |
| Stroop incongruent time (ms) | 1415.7 (261.97) | 1260.47 (223.87) | 1380.28 (164.95) | 1121.66 (91.69) |
| Stroop effect (ms) | 133.94 (169.10) | 197.1 (114.18) | 126.99 (137.56) | 166.4 (86.06) |
| Stroop congruent correct (%) | 89.76 (12.24) | 97.47 (4.37) | 89.38 (14.22) | 91.7 (22.91) |
| Stroop incongruent correct (%) | 81.73 (14.67) | 91.84 (11.05) | 73.33 (26.66) | 87.27 (28.70) |
| Stroop overall correct (%) | 83.57 (12.24) | 93.21 (9.21) | 77.78 (24.67) | 88.61 (26.75) |
| 3-back reaction time (ms) | 1123.01 (236.73) | 958.06 (332.66) | 1119.08 (347.56) | 822.34 (251.52) |
| 3-back overall correct (%) | 68.57 (14.92) | 75.71 (23.35) | 63.89 (22.19) | 78.89 (9.28) |

Source: Choi et al., BMC Neurol. 2024;24:106, Additional Tables 5–6 (main reference 80). Values are mean (SD), reproduced as tabulated. Randomized arms contained 7 ketogenic and 9 standard-diet participants; test-specific denominators were not supplied. The randomized comparison lasted one week. Both groups subsequently received a ketogenic diet, so week-3 outcomes do not estimate a randomized dietary contrast. Comparative cognitive effect estimates and confidence intervals were not reported. The reported Stroop-effect values are retained and are not recalculated by subtracting the displayed latency means.

## Table S6. Hajhashemy 2026 Cognitive Outcomes

Values are mean (standard error, SE), except p values. Both outcomes were secondary. Each intention-to-treat arm contained 46 participants.

| Measure and statistic | Saffron (n = 46) | Placebo (n = 46) | Published between-group comparison |
|---|---|---|---|
| AMT baseline | 8.10 (0.31) | 8.10 (0.30) | Baseline p = 0.99 |
| AMT week 12 | 8.95 (0.21) | 8.17 (0.31) | Change contrasts below |
| AMT change | +0.84 (0.22) | +0.06 (0.30) | Crude +0.78 (95% CI 0.03 to 1.53); baseline-adjusted +0.78 (95% CI 0.16 to 1.40), p = 0.01 |
| AMT within-arm p | <0.001 | 0.83 | Within-arm tests do not estimate the treatment effect |
| PDQ-39 cognitive domain baseline | 25.40 (3.14) | 29.34 (3.07) | Baseline p = 0.37 |
| PDQ-39 cognitive domain week 12 | 19.30 (3.04) | 30.38 (3.44) | Change contrasts below |
| PDQ-39 cognitive domain change | −6.10 (2.32) | +1.03 (2.36) | Crude −7.14 (95% CI −13.73 to −0.55); baseline-adjusted −8.07 (95% CI −14.39 to −1.75), p = 0.01 |
| PDQ-39 cognitive domain within-arm p | 0.01 | 0.66 | Within-arm tests do not estimate the treatment effect |

Source: Hajhashemy et al., Food Funct. 2026;17:889–901, Tables 4–5 (pp. 895–896), main reference 95, doi:10.1039/D5FO01924A. AMT is a 10-item objective screen (higher is better); the interview-administered PDQ-39 cognitive-impairment domain is patient-rated (0–100; lower is better) and is not an objective cognitive-performance measure. Adjusted contrasts used ANCOVA with baseline values; no multiplicity correction was reported. All 92 randomized participants were included using expectation–maximization imputation assuming missingness at random, with baseline characteristics, treatment allocation and available outcomes in the model. Eighty-four completed follow-up (43/41). Published changes and contrasts are retained as printed despite small differences from subtraction of the rounded arm means. CI, confidence interval.
